# A programmable modular robot for the synthesis of molecular machines

**DOI:** 10.1016/j.chempr.2025.102504

**Published:** 2025-03-31

**Authors:** Robert Rauschen, Jean-François Ayme, Bartosz M. Matysiak, Dean Thomas, Leroy Cronin

**Affiliations:** 1 Advanced Research Centre, University of Glasgow, 11 Chapel Lane, Glasgow G11 6EW, UK; 2 Lead contact

## Abstract

The assembly of molecular nanomachines using atomically precise manipulations promises to enable nanotechnology with unprecedented architectural features and exquisite functional properties. However, this future is critically limited by the ability to autonomously manufacture nanomachines, with current efforts being heavily labor intensive. A system is needed to program and assemble matter under digital control, unifying molecular nanotechnology and macroscale chemical processes. Herein, we present a universal chemical robotic synthesis platform (Chemputer) that produces functional molecular machines. By integrating autonomous feedback through on-line NMR and liquid chromatography, a divergent four-step synthesis and purification of molecular rotaxane architectures are achieved. The synthetic sequence averaged 800 base steps over 60 h, affording products on an analytical scale for feasibility studies. While standardizing rotaxane synthesis enhances reliability and reproducibility, our workflow addresses two bottlenecks in autonomous synthesis: yield determination (via on-line ^1^H NMR) and product purification via multiple column chromatography techniques (silica gel and size exclusion).

## In brief

The Chemputer, a universal chemical robotic platform, automates the synthesis of molecular machines, addressing the labor-intensive nature of current methods. By integrating on-line NMR and liquid chromatography within the XDL programming language, complex syntheses are standardized, improving reproducibility and efficiency. As a demonstration, we report the autonomous synthesis of [2]rotaxanes with real-time monitoring for reaction control and purification. By streamlining key challenges, the Chemputer enables broader automation in chemistry, freeing researchers to pursue more ambitious and exploratory work.

## Graphical Abstract

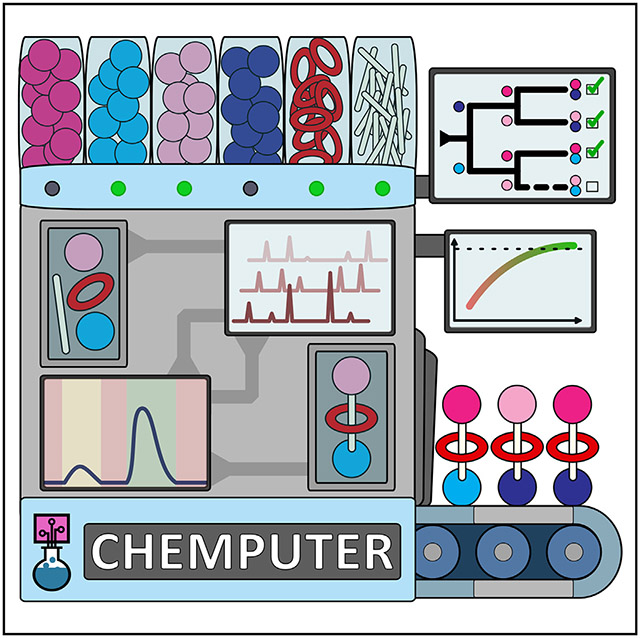

## INTRODUCTION

Automated machines are fundamental to the functioning of both living organisms and our modern societies. In biology, macromolecular machines power all aspects of life, from transporting cargos (e.g., myosin and kinesin) to fabricating nanostructures (e.g., ribosome) to catalyzing chemical reactions (e.g., ATP synthase).^1,2^ At the macroscopic scale, machines enhance human potential by automating tasks, expediting data processing and communication, and by expanding our reach and capabilities.^3-7^ In both of these instances, a fundamental principle emerges of machines assisting and assembling other machines.^8,9^

The fields of supramolecular and systems chemistry have advanced toward this concept. Inspired by the prowess of biological macromolecular machines, chemists have devoted decades to the realization of artificial molecular machines^8-13^— broadly defined as small molecules in which molecular-level motion is harnessed to accomplish a practical task.^14-17^ As movement on the nanoscopic scale is governed by Brownian motion, the unique properties of mechanically interlocked molecules, encompassing rotaxanes, catenanes, and knots, have been critical motifs in the advancement of artificial molecular machines.^10-12^ Rotaxanes in particular utilize bulky axle-terminating “stoppers” to limit the degrees of freedom of a threaded macrocycle, permitting the precise molecular-scale control of motion relative to the axle in response to external stimuli.^18^ This property has been fundamental to a range of seminal molecular machines, including molecular switches, switchable catalysts, molecular assembly lines, and molecular pumps.^13^

**Figure 1.**
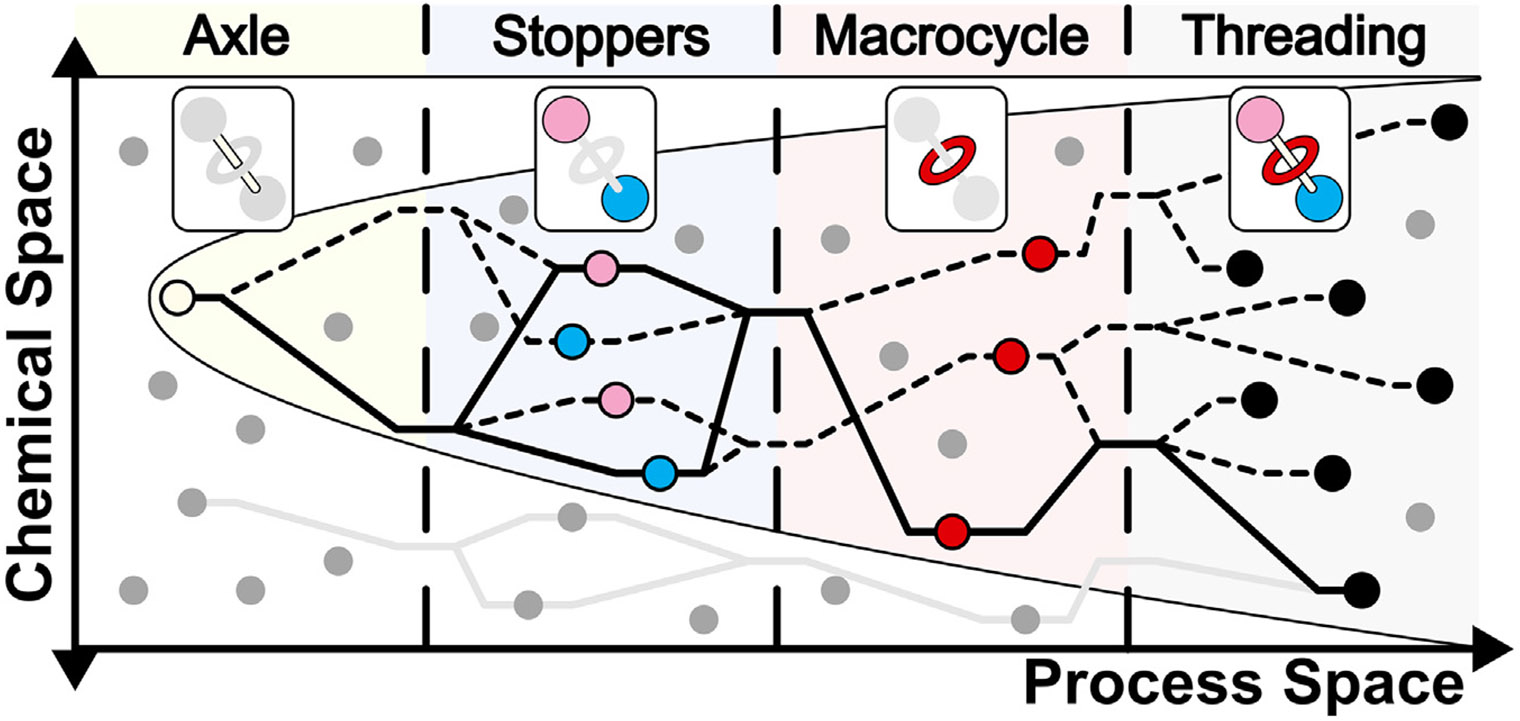
Exploration of rotaxane-based chemical and process space Autonomous platforms (black paths) execute dynamic procedures and diverge to explore alternate routes in chemical and process space to synthesize compounds more efficiently. Conventional automated platforms (gray path) execute fixed, linear sequences through process space at the expense of unexplored synthetic conditions, routes, or compounds. This exploratory approach is illustrated with [2]rotaxanes but is generally applicable to alternate artificial molecular machine architectures.

Despite momentous progress in the field of rotaxane-based artificial molecular machines, their production is still a laborious process requiring skilled chemists. The first challenge is in the optimal design of these systems owing to the vast number of parameters that define the combinatorial chemical space. Critical opportunities for application-dependent activity can be tuned by varying the axle (and constituent binding sites), stoppers, and macrocycles (Figure 1). Ideal properties are determined from either preexisting literature, simplified model studies, and/or through trial and error, the latter of which being extremely time consuming. Exploring more complex, unknown systems will only be feasible if there exists a versatile and robust toolkit of easily and reliably accessible molecular machines at our disposal.^19^

The second challenge is in the efficient synthesis, assembly, and evaluation of these systems. Automating synthetic chemistry is a multifaceted problem due to the diversity of organic reactions and the complexity of multistep syntheses. Thus, it is nontrivial to create a universal robotic platform for generalized organic chemistry.^20-22^ Successful applications of automated, batch synthesis are limited to iterative syntheses of specific oligomeric molecules (e.g., oligopeptides,^23^ oligonucleotides,^24^ oligosaccharides,^25^ and *N*-methyliminodiacetic acid (MIDA) boronate building blocks^26^), where the successive repetition of a small number of robust reactions facilitates automation. Traditionally, these platforms are programmed to execute a sequence of predefined steps in process space, irrespective of dynamic properties such as conversion or reaction yields, which are crucial for closed-loop exploration. Consequently, such systems are restricted to hard-coded, linear syntheses, often manually interrupted. An autonomous chemistry platform, on the other hand, has the capability to execute multiple syntheses in series or in parallel and scale subsequent reactions dynamically to explore chemical and process space more efficiently (Figure 1).

As the interest, design, and evolution of supramolecular systems increase, the final challenge to overcome is the lack of standardization of literature procedures in the field. In recent years, however, significant progress has been made toward creating more universal and programmable chemical synthesis robots.^27-34^ With these systems, classical multistep synthetic protocols are translated into standardized—yet amenable—digital synthetic steps that are executed by robotic hardware, effectively formalizing the syntheses of molecules.^35,36^ The digital protocol produced during this process can be published, versioned, and transferred to other machines, enhancing the reproducibility and reliability of the reported syntheses.^37^ So far, a wide range of contemporary organic reactions, including solidphase peptide, cross-coupling reactions, heterocycle formations, functional group interconversions, and multicomponent reactions, have been successfully demonstrated alongside the synthesis of pharmaceuticals (e.g., rufinamide and lidocaine) or expensive reagents (e.g., diazirines, Dess-Martin periodinane, and fluorinating agent AlkylFluor).^33,34,36^

**Figure 2.**
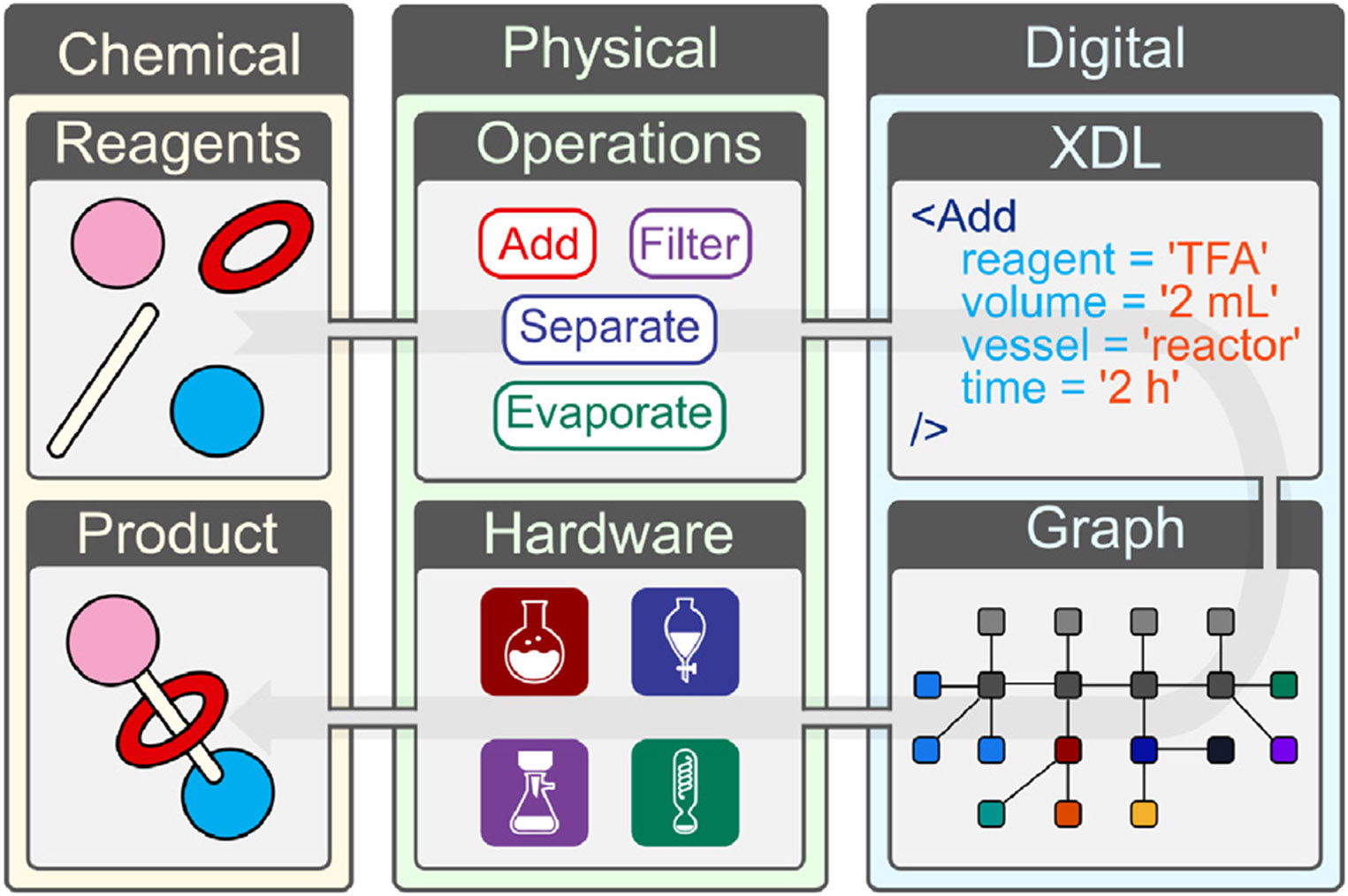
General Chemputer-based workflow for the synthesis of rotaxane-based molecular machines Chemical reagents are subject to physical unit operations as encoded by the digital chemical description language (XDL), mapped onto a digital graph representation of the physical hardware, which then synthesize the chemical product. By use of abstract XDL unit operations bound to executable code, the synthesis can be simulated on a digital representation of the Chemputer and can be achieved using its physical hardware. This workflow is specific for [2]rotaxanes but can be generalized for alternate artificial molecular machine architectures.

To date, no supramolecular systems, let alone mechanically interlocked species, have been digitally programmed, synthesized, and purified by autonomous means. Herein, we report the autonomous synthesis of four rotaxane-based molecular machines by digitizing their reaction procedures, using the universal chemical description language XDL, and executing these digital procedures on the automated synthesis Chemputer platform (Figure 2). The rotaxanes were diversified with a variety of stoppers and stations, demonstrating the versatility of the approach. The resulting multistep procedure, including reductive amination, nitro reduction, threading, and capping, comprised over 800 base steps (on average) executed continuously over 60 h of platform runtime.

## RESULTS AND DISCUSSION

**Figure 3.**
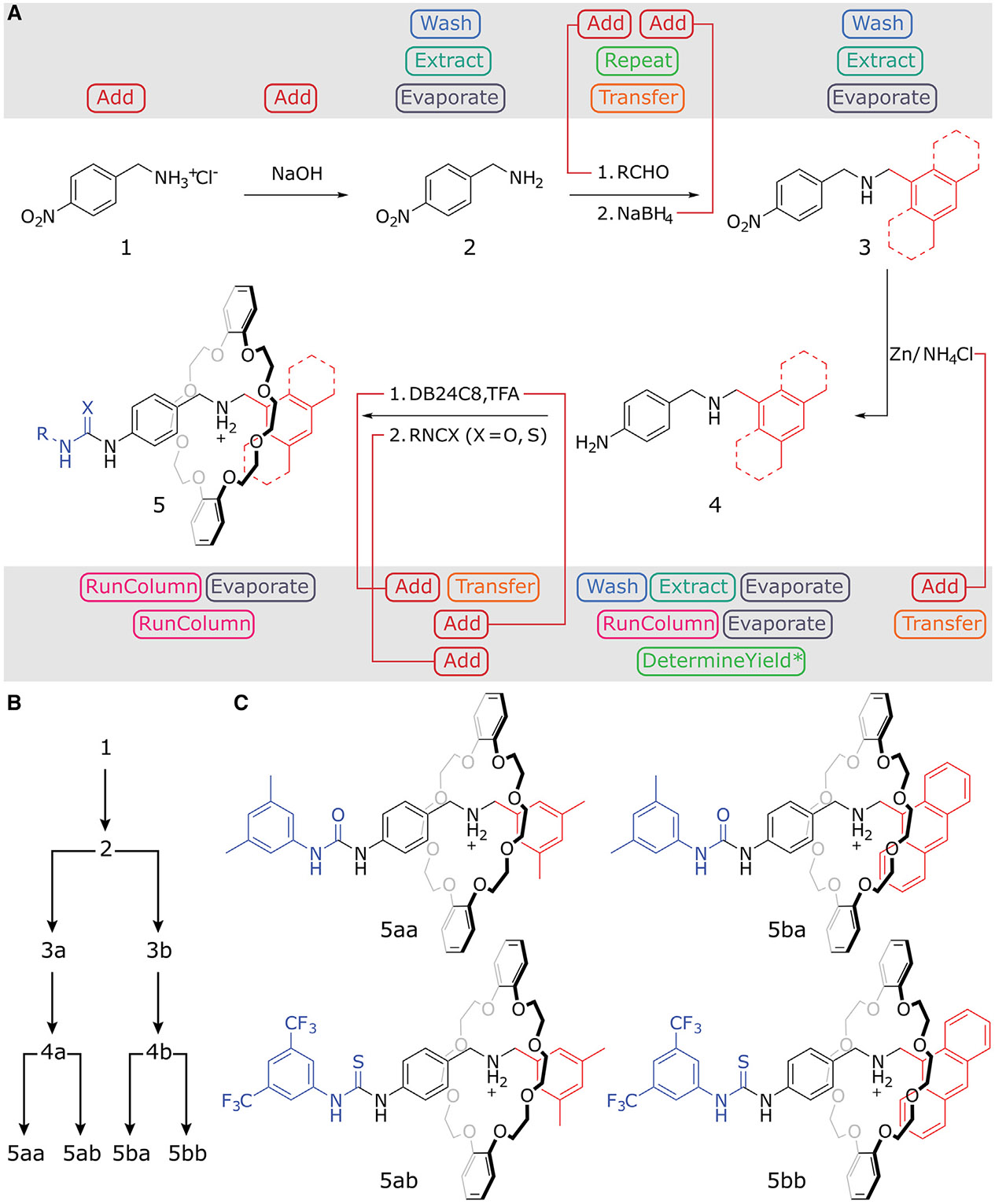
Divergent, automated synthesis of rotaxanes (A) Programmed four-step synthetic route autonomously executed by the Chemputer encompassing reductive amination, nitro reduction, threading, and capping of the rotaxane. (B) Branching synthetic route, see also Scheme S1. (C) Rotaxanes synthesized in this study.

[2]Rotaxanes were selected as a scaffold for our study, as this motif has proven effective in producing molecular machines for a broad range of applications.^15,18,38^ Starting from 4-nitrobenzylamine hydrochloride **1**, a range of [2]rotaxane-based machines could be obtained via a four-step synthesis (Figure 3A): (1) the first stopper and station could be introduced through the free-basing of **1** followed by the reductive amination of the resulting (unstable) amine **2** in the presence of a variable aldehyde, (2) the nitro substituent of dibenzylamine **3** is then reduced to reveal the arylamine of half-capped axle **4**, (3) a dibenzo-24-crown-8 macrocycle (**DB24C8**) threads onto **4** via the selective protonation of the half-capped axle dibenzylamine^39,40^ with two equivalents of trifluoroacetic acid (TFA), and (4) finally, the rotaxane could be fully capped via (thio)urea formation, simultaneously installing the second station for the macrocycle to shuttle between.^41^ Using 9-anthracenecarboxaldehyde or 3,5-dimethylbenzaldehyde as precursors for the first stopper and 3,5-dimethylphenyl isocyanate or 3,5-bis(trifluoromethyl)phenyl isothiocyanate as capping reagents, four molecular machines could be obtained via a divergent synthesis approach (Figure 3B), demonstrating the versatility of our design. These four reagents were chosen to cover known literature examples of crown ether-based molecular machines utilizing anthracene photoinduced electron transfer (PET) sensing and/or (thio)urea hydrogen-bonding catalysis.^38,42,43^

**Figure 4.**
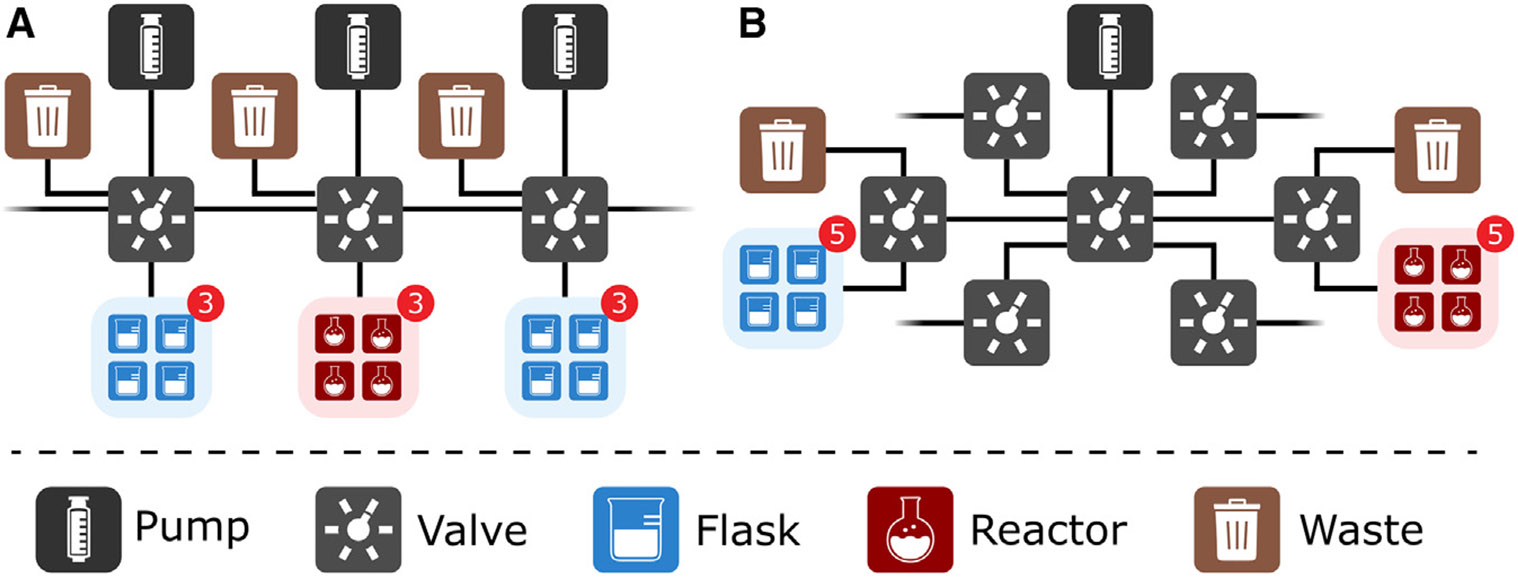
Connectivity of the Chemputer (A) The standard linear backbone affords lucid connections and movement paths at the cost of fewer ports available for reagents and modules. (B) The daisy-chained backbone accommodated the extended needs of this synthetic route while in a hardware-efficient manner. The numbers in the red circles indicate the number of items (reactors/flasks) that are connected to the respective valve.

The standard Chemputer backbone consists of six pump-valve pairs, connected in series via polytetrafluoroethylene (PTFE) tubing.^34^ Each valve comprises six horizontal ports (of which one is reserved as a default waste connection) and one vertical port (for the paired pump). After accounting for the abovementioned connections, 20 valve ports remain available on the backbone to connect reagents, reactors, and modules such as the rotary evaporator. This default configuration is sufficient to execute most synthetic workflows; however, if necessary, the backbone can be extended *ad infinitum* by adding extra valve-pump pairs, offering three additional valve ports per pump-valve pair (Figure 4A). To accommodate the necessities of our multistep, divergent synthesis, however, a more space- and hardware-efficient organization of the Chemputer backbone needed to be developed. Accordingly, auxiliary valves were connected to the backbone valves in a “daisy-chained” configuration (Figure 4B). This arrangement increased the number of available ports on the Chemputer backbone for reagents and modules from three to five per valve, and consequently, the entire platform utilized only four pumps and ten valves (versus twelve pump-valve pairs if the standard Chemputer backbone configuration was utilized).

The implementation of daisy-chained valve configurations required additional considerations for the pathfinding algorithms (those responsible for determining the optimal path among the various modules and reagents of the Chemputer during the execution of synthetic instructions based on the user-defined 2D graph representation of the backbone). First, default waste containers needed to be connected to the daisy-chained auxiliary valve instead of the backbone primary valve to allow for cleaning of the daisy-chained auxiliary valve connection (Figure 4B). Likewise, pathfinding algorithms then needed to identify all “end-wastes” on the branched arrangement and to transfer cleaning solvents correctly to all connections. Second, priming of the tubing prior to the addition of a reagent needed to be adapted. This process is crucial to avoid substance loss due to the dead volume between the reagent flask and the aspiration pump. An updated pathfinding algorithm was implemented, which ensured that there is no overlap between the primed line and one that is used to discard excess priming volume. Other-wise, priming would be negated as the daisy-chained connection would be emptied while transferring the excess to the nearest waste.

**Figure 5.**
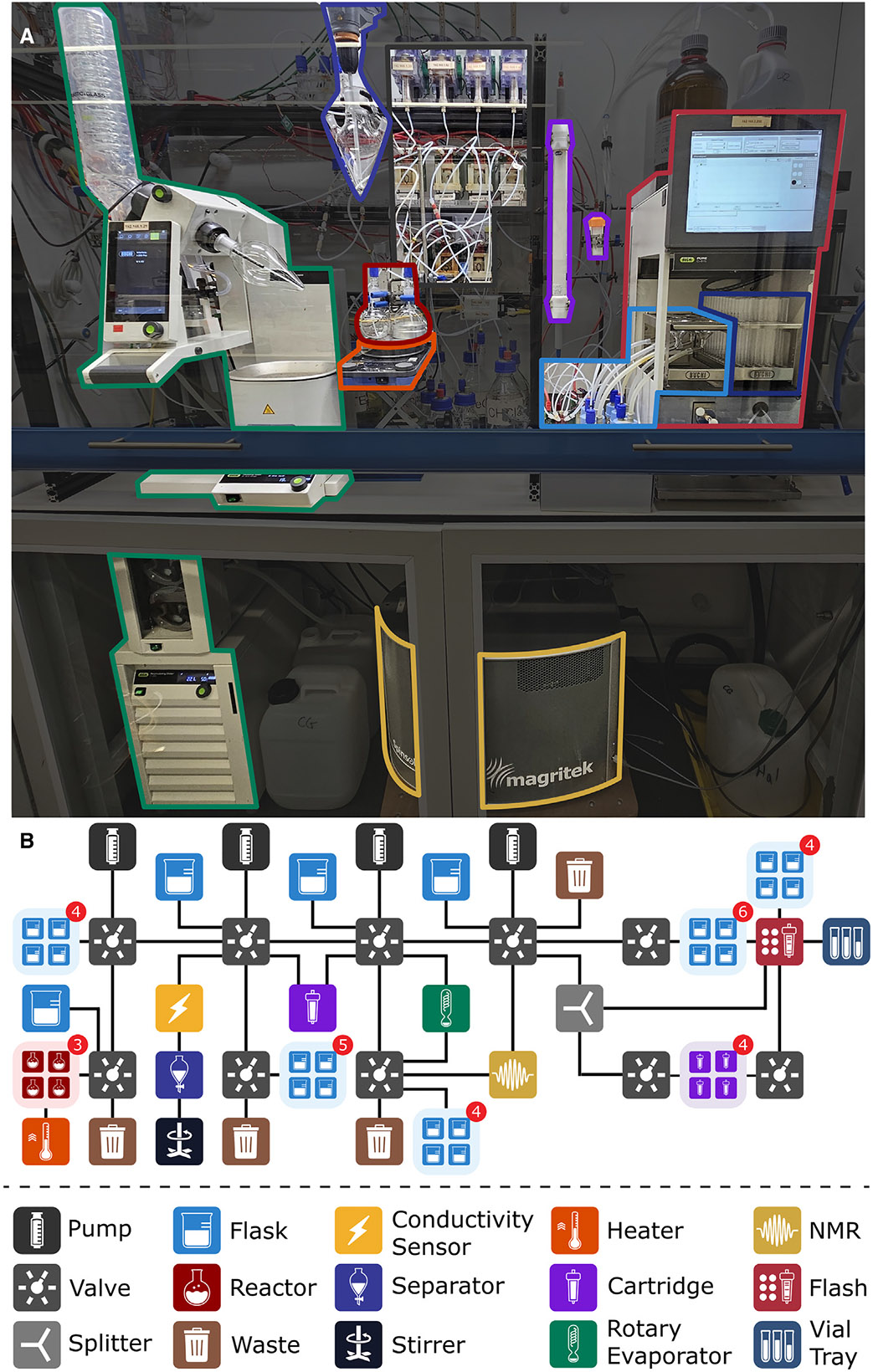
Physical and graphical representation of the Chemputer (A) The modular platform comprised readily available, commercial devices. (B) The graphical representation of the physical hardware. The numbers in the red circles indicate the number of items (reactors/flasks/cartridges) that are connected to the respective valve.

Realizing a multistep synthesis as a continuous, automated workflow required the following: (1) a feedback mechanism for autonomous reaction monitoring and yield determination, implemented via on-line ^1^H NMR, and (2) autonomous product purification via multiple column chromatography techniques, namely, silica gel and size-exclusion column chromatography. Owing to the modularity of the Chemputer platform, these functionalities could be achieved by directly integrating readily available commercial devices and process analytical tools (PATs) into the workflow (Figure 5). Automated purification of the rotaxanes within the Chemputer setup was achieved with a Buüchi Pure C-815 Flash chromatography system, whose integration into the XDL software environment was prototypically described by Rohrbach et al.^34^ Modifications to the original configuration improved the injection protocol utilizing the Chemputer backbone by priming all tubing involved with eluent—including the adjacent column bypass—to avoid column cracking by injection of air from the tube’s dead volume. Physical adjustments included the usage of a tubing splitter, as depicted in Figure S2, to enable the improved sample injection. Subsequent dissolution of the crude material inside the rotary evaporator flask, followed by wet loading onto the column and then rinsing with minimal solvent, maximized mass recovery and minimized peak broadening on the chromatography system. Multiple columns were mounted on a carousel for use throughout the synthetic procedure, removing the need for human intervention. The elution was monitored by UV absorption at 220 or 254 nm. The collected fractions were directed to vessels connected to the Chemputer backbone, allowing the purified products to be seamlessly utilized in subsequent steps of the automated synthetic workflow. The connectivity of the individual components of the chromatography setup is displayed in Figure S4. The fraction with the largest peak area was assumed to be the product fraction and was transferred from the fraction vessel(s) to the rotary evaporator for further analysis. Only in the case of the final purification via size-exclusion chromatography were fractions collected in the test tube tray shown in Figure S2 to collect the minimal sample size that minimized co-elution and maximized sample purity. Manual direct injection mass spectrometry was then used to aid the identification and characterization of the final product fractions. The parameters for operating the mass spectrometer are provided in Table S1. This process could also be fully automated by using more advanced, mass-directed flash chromatography systems.

While prior work had utilized benchtop NMR apparatus,^44,45^ its integration in the codebase was context dependent. Therefore, a general protocol for analysis, including cleaning of required liquid paths, priming, and sampling of the flow cell fitted into the apparatus, as well as the actual NMR measurement, had to be developed for this work. The flow cell was implemented as a “cartridge” in the XDL software environment to ensure that liquid transfers were interpreted to go through the flow cell instead of terminating there. The sampling protocol, enabling the transfer of an aliquot from any liquid-containing vessel, was implemented as a XDL step specific to the Chemputer platform but agnostic about the analysis method. This design allows for the sampling protocol to be used for other flow cell-based spectrometers (i.e., on-line infrared [IR] and ultraviolet-visible [UV-vis]) in future applications within the Chemputer platform. It also grants end-users the flexibility to predefine an appropriate NMR sequence with method-specific properties. Shimming on the sample was explicitly included as a separate NMR experiment before acquiring the spectra when needed.

**Figure 6.**
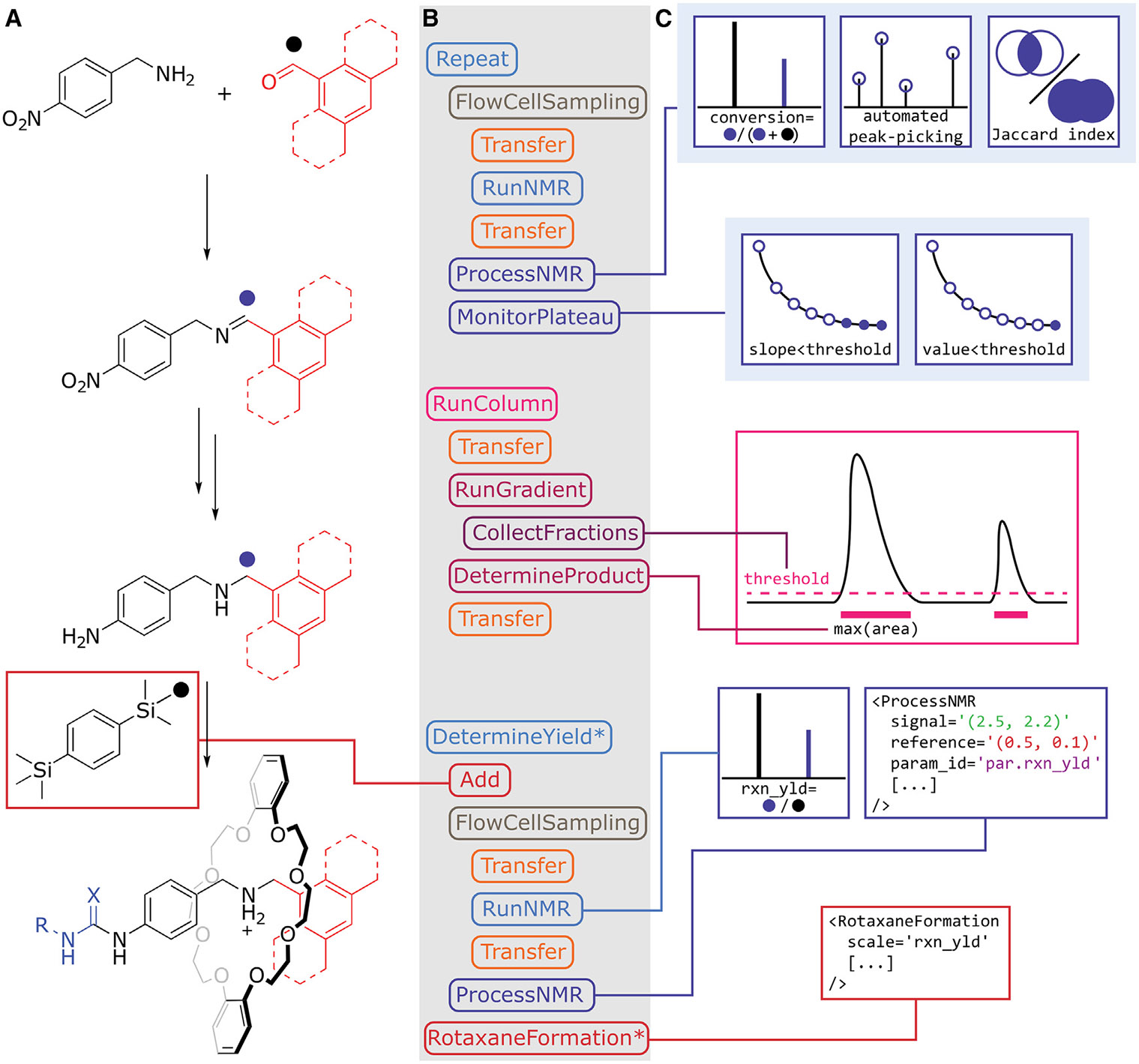
Feedback-controlled steps for the autonomous synthesis of rotaxanes (A) Sections of the generalized synthetic route that required dynamic parameters to afford rotaxane **5**. (B) Definition of feedback-controlled XDL steps and their corresponding sub-steps. (C) Methodologies for assessing and adapting to the inherent variation between reactions dynamically. The runtime to afford one rotaxane was approximately 60 h, and the remaining three were purified within 72 h, owing to the parallel nature of the synthesis but queued usage of the purification equipment. Each rotaxane (**5aa, 5ab, 5ba,** and **5bb**) was chromatographically pure (16%, 63%, 15%, and 8% yield of the final step after threading and capping, respectively). We report more details on the reproducibility and reliability of the automated synthesis in Table S2.

Using ^1^H NMR as an autonomous feedback mechanism for reaction monitoring required additional development of the XDL software environment (Figure 6). Determining the plateau of a reaction based on conversion was initially proposed, mimicking the usual strategy employed manually by chemists.^46^ However, applying this approach in the case of a multistep process is impractical, as it necessitates *a priori* knowledge of the chemical shifts of key diagnostic NMR peaks before conducting the experiment. The limitation of this method hinders its use in a discovery-oriented, exploratory setting. Alternatively, automated peak picking, which tracks changes as a function of reaction progression, removes the need for specifying chemical shifts. Diagnostic signals are defined as peaks characteristic for a compound but also intense enough and not overlaid by solvent signals to calculate meaningful integrals on the benchtop NMR machine. Identification of the diagnostic signals by this method enables automated conversion calculation and reaction endpoint detection in the next iteration of the reaction; however, this approach is susceptible to errors, for instance, in cases where migrating signals of exchangeable protons are present or when the splitting patterns of signals change during the reaction.

Considering the aforementioned limitations, an alternate metric based on the Jaccard similarity index was proposed, aiming to add further autonomy to the process. The Jaccard index is used to quantify the degree of overlap between two sets, with a higher index value indicating a greater similarity. As the reaction progresses, some of the signals grow, some shrink, and some (i.e., exchangeable protons) shift. However, the sum of the area under all signals in the ^1^H NMR spectrum should remain the same (assuming that no reaction mass is lost from the solution through, for example, solvent evaporation or precipitation). Thus, the extent to which the reaction deviates from its initial state at a given time is found by comparing the integration ensemble of all signals in a^1^H NMR spectrum with the initial ^1^H NMR spectrum. Eventually, as the reaction nears completion, no further changes to the spectrum should be observed, and so the Jaccard index should settle. The Jaccard index can therefore serve as a proxy for evaluating the proximity of a reaction to equilibrium when employing an automated and autonomous monitoring process. Overall, we provide a toolkit to investigate the NMR properties of the reaction being examined and combine that information with the chemical knowledge about the reaction to increase the likelihood of successful automated endpoint detection (see NMR monitoring). Having enhanced the software and hardware capabilities of XDL and the Chemputer, respectively, we demonstrated the applicability of the developed technology by autonomously synthesizing four rotaxane-based molecular machines starting from their commercially available building blocks (Figure 6).

As different substrates exhibit different physicochemical properties and reactivity, using a standardized protocol for a general synthesis route would be impossible. At the first diverging point of the route, two different aldehydes were used for the imine formation, followed by reduction with sodium borohydride. As the electrophilicity of aldehydes varies as a function of their structure, the conversion was monitored with on-line NMR to determine the endpoint of the reaction. Reaction monitoring revealed that the imine formation using 9-anthracenecarboxaldehyde proceeds at a drastically slower rate, which was evident by following the conversion calculated by dynamically comparing the imine and the aldehyde signals (see supplemental methods; Figure S5). The reaction time was then manually adjusted in the XDL to ensure the conversion plateaued before automatically triggering the workup protocol. As imine formation was not quantitative, purification via column chromatography on silica gel was required to afford arylamine **4** as a clean product following the reduction of **3**. Using the photo-diode array detectors integrated within the commercial chromatography setup allowed for feedback-controlled collection of fractions and determination of the product-containing fraction, which was subsequently transferred back into the rotary evaporator from the bottles that are connected to the collection tray in Figure S1. While still needing to hardcode column volumes for the elution gradient, the feedback control alleviated the need to specify column volumes for collection.

The final step of the synthesis, rotaxane formation, was dependent on the number of equivalents of TFA added to the reaction mixture. This dependence is to be expected, as the reaction requires a fine balance between protonating the aliphatic amine such that it binds the crown ether and limiting the protonation of the arylamine, as it acts as a nucleophile toward the (thio)isocyanate. In an automated multistep synthesis, managing the stoichiometry of a specific reaction beyond the initial step requires the ability to determine the yield of the preceding step autonomously and automatically (e.g., assessing the yield of the reduction from **3** to **4** for executing subsequent rotaxane formation steps leading to **5**) to adjust the remaining protocol. This essential adjustment, to the best of our knowledge, has not been realized to date in any automated multistep synthetic workflow. To achieve this, on-line ^1^H NMR analysis was employed to allow for the determination of the reaction yield without the need for manual intervention in the automated workflow. Yield determination was achieved by adding a known amount of 1,4-bis(trimethylsilyl)benzene (TMSB) as an internal standard as part of the solvent used to redissolve the product. The autonomous calculation of an NMR yield was performed by comparing its methyl signal against the diagnostic methylene/benzylic signals of the diamine. The determined NMR yield was subsequently utilized to adjust the stoichiometry of the reagents added in the subsequent steps, specifically for the addition of TFA. The inert TMSB standard is then removed during the purification of the final rotaxane by column chromatography (silica gel followed by size exclusion).

### Conclusions

By digitizing reaction procedures using the XDL standard and executing these protocols on the Chemputer, the first automated and autonomous synthesis of rotaxane-based artificial molecular machines was achieved. The rotaxanes themselves demonstrate a proof of principle. With an established and verified XDL, using automated reaction endpoint determination, on-line yield calculation, and automated chromatography, the synthetic route can be modified with alternate stoppers, macrocycles, and stations to permit an even larger library of compounds for further application testing and optimization. The capabilities of this platform are not limited to [2]rotaxanes, but they could be extended to a broader range of molecular machine scaffolds, including rotors, motors, and ratchets.

The modular, architectural design presented here can be utilized for a robotic factory that operates across scales, permitting the rapid exploration and small-scale preparation of a library of related compounds. Importantly, this can be automatically scaled or numbered up to the batch synthesis of gram quantities of an identified compound of interest without any changes to the robotic architecture. This means that arrays of robotic synthesis modules can be used for the simultaneous exploration or exploitation of chemical space, enabling the manufacturing of molecular machines on an industrial scale. Thus, it is expected that future chemists will be able to focus on the interesting applications of these machines in catalysis and material and systems chemistry.

## METHODS

All details regarding the experimental methods and procedures can be found in the supplemental methods as well as in Figures S1-S5, Scheme S1, Tables S1 and S2, and Spectra S1-S12.

## Supplementary Material

Supplementary Material

## Highlights

- Automation of time-consuming [2]rotaxane synthesis with minimal human intervention
- On-line spectroscopy dynamically adjusts process conditions with real-time feedback
- The chemical description language XDL affords synthetic reproducibility

## THE BIGGER PICTURE

The precise assembly of molecular machines can revolutionize nanotechnology by creating structures with unmatched complexity and functionality. However, current synthetic methods are time and labor intensive, limiting scalability and innovation. We address this challenge using the Chemputer, a universal chemical robotic platform. By integrating on-line feedback from NMR and liquid chromatography within the chemical programming language XDL, the Chemputer standardized and autonomously executed complex syntheses, improving reproducibility and efficiency. As a demonstration, we report the synthesis of a series of [2]rotaxanes, with real-time monitoring ensuring controlled reaction progression and purification. By addressing and streamlining these key challenges in automation, the Chemputer provides a more reliable approach to constructing advanced molecular architectures. This platform represents a step toward broader automation in chemistry, where programmable synthesis could improve access to complex molecules across various fields. With routine operations automated, researchers are freed from the constraints of repetitive, manual experimentation, enabling more ambitious and exploratory work that may lead to unexpected discoveries.
