# Supplementary material for: A programmable modular robot for the synthesis of molecular machines
Source: Chem. Author manuscript; Available in PMC 2025 Aug 29. (PMC12393038; doi:10.1016/j.chempr.2025.102504)
Supplement: Supplementary Material [file NIHMS2078890-supplement-Supplementary_Material.pdf]

Chem, Volume *11*

Supplemental information

A programmable modular robot for the synthesis of molecular machines

Robert Rauschen, Jean-François Ayme, Bartosz M. Matysiak, Dean Thomas, and Leroy Cronin

Contents

1 General Experimental Section 2

1.1 Abbreviations 2

1.2 Materials 2

1.3 NMR 2

1.4 MS 2

1.5 Chromatography 3

2 Software 5

3 Hardware 6

4 NMR monitoring 6

5 Synthesis 7

5.1 Synthetic Outline 7

5.2 Automated Procedures & Characterisation Data 8

5.3 ^{1}H and ^{13}C NMR Spectra 24

6 Reliability & Reproducibility 30

7 References 31

1 General Experimental Section

1.1 Abbreviations

COSY – Correlation Spectroscopy; **DEPT** – Distortionless Enhancement by Polarization Transfer; **ESI** – Electrospray Ionization; **HMBC** – Heteronuclear Multiple-Bond Correlation Spectroscopy; **HSQC** – Heteronuclear Single Quantum Coherence Spectroscopy; **MS** – Mass Spectrometry; **NMR** – Nuclear Magnetic Resonance; **NP** – Normal Phase Chromatography; **ROESY** – Rotating-Frame Nuclear Overhauser Effect Correlation Spectroscopy; **RPM** – Revolutions Per Minute; **RT** – Room Temperature; **SEC** – Size Exclusion Chromatography; **TLC** – Thin Layer Chromatography; **THF** – Tetrahydrofuran; **UV** – Ultraviolet; **XDL** – Chemical Description Language.

1.2 Materials

Unless stated otherwise, reagents were obtained from commercial sources and used without purification. Room temperature (r.t.) reactions were carried out between 21 – 23 °C.

1.3 NMR

^{1}H NMR spectra were recorded on either a Bruker Avance III HD 600 (MHz) NMR spectrometer equipped with a cryoprobe at 300 K or a Magritek Spinsolve 60 (MHz) Ultra at 298 K. Chemical shifts are reported in parts per million (ppm) from high to low frequency using the residual solvent peak as the internal reference ($CDCl_3$ = 7.26 ppm, CD_2Cl_2 = 5.32 ppm, and CD_3CN = 1.94 ppm). All ^{1}H resonances are reported to the nearest 0.01 ppm. The multiplicity of ^{1}H signals are indicated as: s = singlet; d = doublet; t = triplet; quint = quintet; m = multiplet; br = broad; or combinations of thereof. Coupling constants (*J*) are quoted in Hz and reported to the nearest 0.1 Hz. Where appropriate, averages of the signals from peaks displaying multiplicity were used to calculate the value of the coupling constant. ^{13}C NMR spectra were recorded on the same Bruker spectrometer with the central resonance of the solvent peak as the internal reference ($CDCl_3$ = 77.16 ppm, CD_2Cl_2 = 53.84 ppm, and $\underline{C}D_3CN$ = 1.32 ppm). All ^{13}C resonances are reported to 0.01 ppm to aid in the differentiation of closely resolved signals. NMR spectra were digitally processed (phase and baseline corrections, integration, peak analysis) using MestReNova 14.3. ^{1}H and ^{13}C NMR assignments were made using 2D-NMR methods (DEPT, COSY, ROESY, HSQC, HMBC). Fully characterized compounds were chromatographically homogeneous.

1.4 MS

High resolution ESI mass spectra were obtained by direct injection into a Bruker MaXis Impact quadrupole time-of-flight mass spectrometer with an electrospray source, operating exclusively in positive mode. The instrument parameters are detailed in Table S1. The instrument was regularly calibrated using Agilent ESI-L Low Concentration Tuning Mix. Data was analysed using the Bruker Data Analysis v4.1 software suite.

Table S1: Mass Spectrometry Parameters

**Parameter**	**Value**
Dry gas temperature	200 °C
Ion polarity	positive
Voltage at capillary tip	4500 V
Voltage at end plate offset	500 V
Nebuliser pressure	1.6 bar

Dry gas flow	8.0 l/min
Funnel 1 radio frequency	400 $V_{peak-to-peak}$
Funnel 2 radio frequency	400 $V_{peak-to-peak}$
In-source collision-induced dissociation energy	0 eV
Hexapole radio frequency	100 $V_{peak-to-peak}$
Ion energy	5.0 eV
Collision energy	5 eV
Collision cell radio frequency	200 $V_{peak-to-peak}$
Transfer time	63.5 µs
Pre-pulse storage time	1.0 µs
Mass range	50 – 2000 m/z

1.5 Chromatography

Automated flash column chromatography was carried out using a Büchi Pure C-815 Flash Chromatography system with commercially available cartridges. Size exclusion chromatography utilised the same hardware with a Diba Omnifit® EZ SolventPlus™ Chromatography Column containing Bio-Beads S-X3 support beads as the stationary phase. Analytical TLC was performed on precoated silica gel plates (0.25 mm thick, 60 F254, Merck, Germany) and visualized using both short and long waved ultraviolet light in combination with standard laboratory stains (acidic potassium permanganate, acidic ammonium molybdate).

To interplay with the rest of the Chemputer setup, the Büchi system was connected to the backbone of the rig as demonstrated in Figure S1. First, four solvent reservoirs were connected to ports A to D of the chromatography node C815. Next, a cartridge carousel was set up using valve_96 as the input valve and valve_35 as the output valve. Between those valves four cartridges were connected so that the Chempiler algorithm can automatically switch between the bypass line, the size exclusion column, and the two silica columns as required. The top ports (port -1) of the two valves were connected to the solvent inlet and outlet of the Büchi system in a circular way so that the eluent can automatically be pumped through the cartridges by the Büchi system. Furthermore, the top port of valve_96 was extended with a 4+1-way splitter as shown in Figure S2 to allow for an additional connection to valve_85, which is part of the Chemputer backbone so that samples can be injected into the chromatography section using the standard backbone operations. Finally, the Büchi system was equipped with two types of fraction collection vessels. On the left-hand side as shown in Figure S2, a six-port funnel tray was mounted and the outlets were connected to 250 mL Duran bottles as shown in Figure S1, which are labelled as fraction_1 to fraction_6 on the graph and are connected to the Chemputer backbone again to allow retrieval of the product fraction(s) after purification. On the right-hand side, a tray with 65 test tubes (15 mL volume) was mounted to allow for a finer resolution of fractions during the final purification of the rotaxanes via size-exclusion chromatography.

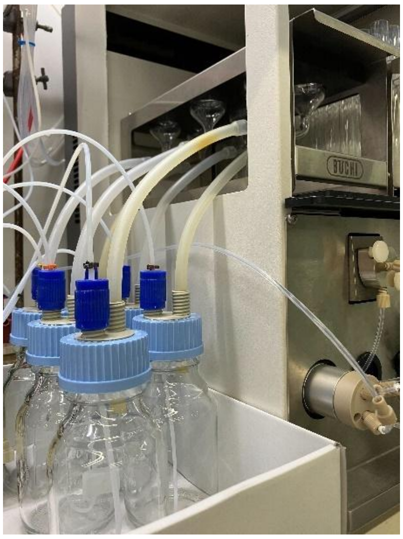

Figure S1*:* Fraction flasks connected to the funnel tray of the Büchi system.

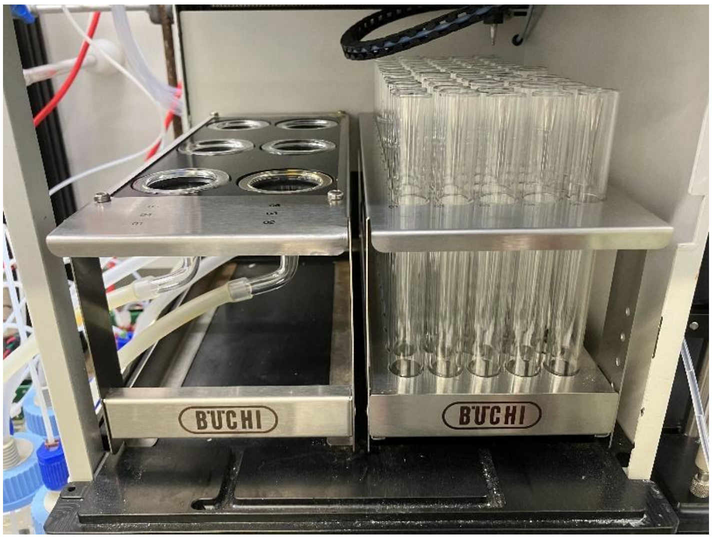

Figure S2: Fraction collectors of the Büchi system. Left: six-port funnel tray, right: 65-vial tray.

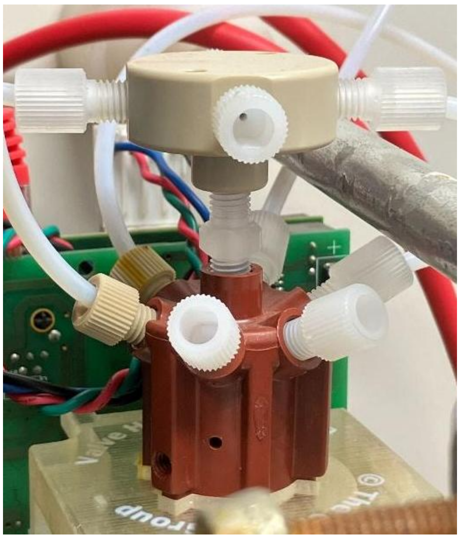

Figure S3: Line splitter to extend the top port of valve_96.

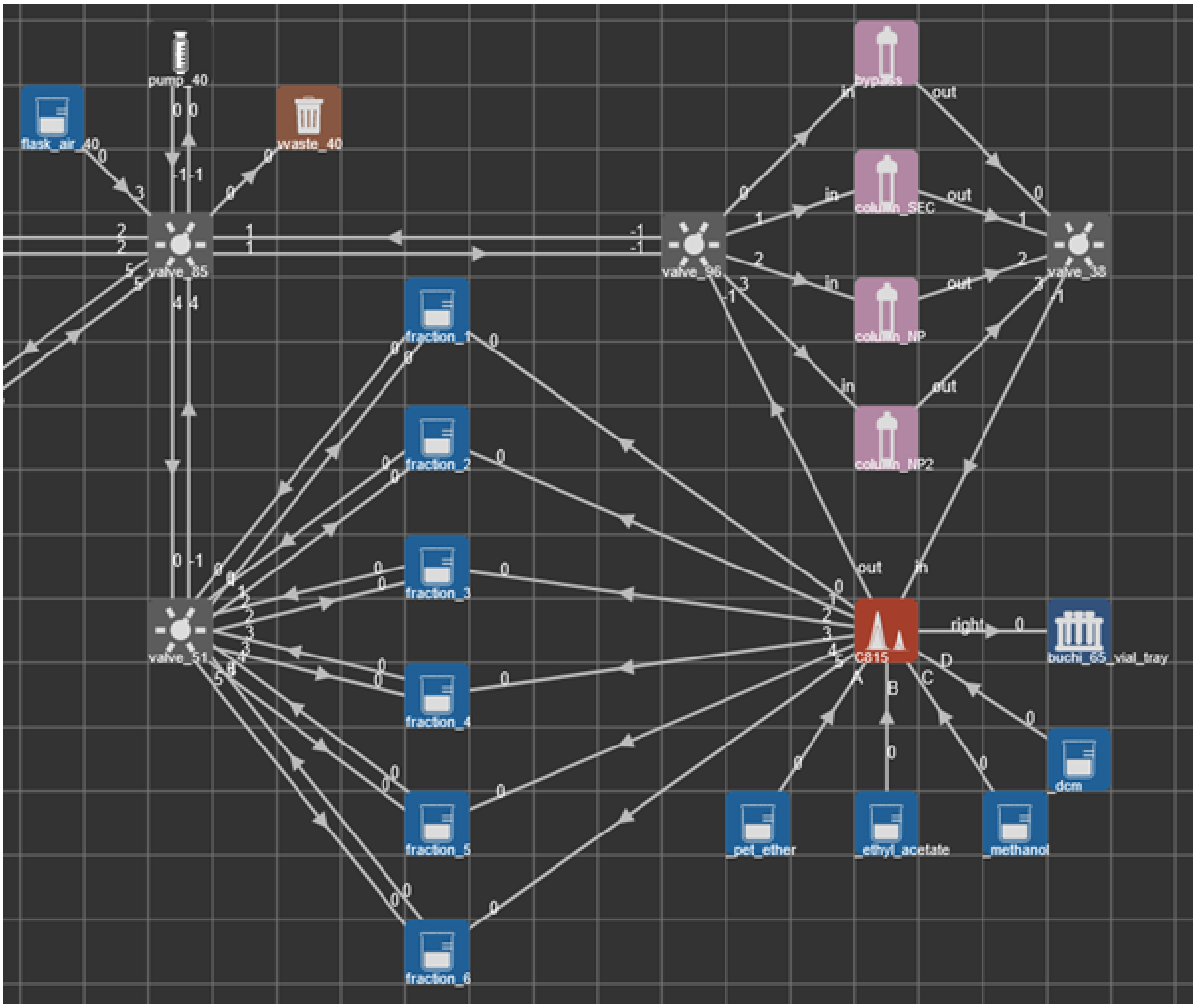

Figure S4: Section of the graph representation of the Chemputer setup illustrating the integration of the Büchi flash chromatography system (node C815) and the cartridge carousel (nodes bypass, column_SEC, column_NP, column_NP2, valve_96, valve_35).

2 Software

All XDL and graph files utilised in this study are presented with this document. In addition, a generic run script that is used to operate the platforms with a python interface is added. The chemical information in the XDL files will be repeated as human readable output here for clarity. Bold sections in the XDL output mean **manual performance by chemists.** If a bolded step is also a numbered step, that step is detailed within the XDL itself, but is still performed manually. Italic phrases in the XDL output are comments to *clarify the intention of the step*.

This is a manual step performed by the chemist, not detailed in the XDL.

1) **This is a manual step performed by the chemist, contained within in the XDL.**
   This is additional information about the above step.

Chemputer graph files (.json) can be opened and edited with the ChemIDE app on:

https://croningroup.gitlab.io/chemputer/graphapp/

The XDL software standard is linked here:

https://croningroup.gitlab.io/chemputer/xdl/standard/index.html

A complete docker image of the whole software stack that is needed to compile, simulate, and execute the XDL protocols can be made available on reasonable request. The exact terms of use for the software are explained on the Cronin Group homepage:

http://www.chem.gla.ac.uk/cronin/media/license/.

3 Hardware

Unless stated otherwise, all hardware utilised is part of the core Chemputer framework extensively described previously. [S1]

4 NMR monitoring

To get a full picture of the reaction progression during the imine formation we monitored the reaction between 4-nitrobenzylamine and 9-anthracenecarboxaldehyde overnight. This allowed us to fully resolve the plateau region as can be seen in Figure S5. In the presented example, we calculated the NMR conversion by dividing the diagnostic product imine signal by the sum of the product imine signal and the substrate aldehyde signal. We extrapolated the plateau as a horizontal line using the data points at the end of the series to get an idea of when the conversion has reached its endpoint within the error margin of the data.

Subsequently, we revisited the data and walked through it point by point, calculating the slope of the data points within a window of five points. As soon as the window lied within the previously defined plateau, we made note of the slope value (1e-4 in this case) and used it as a threshold for slope-assisted endpoint detection in following experiments. This is to ensure that the algorithm still works if the magnitude of change in the NMR spectra changes between experiments.

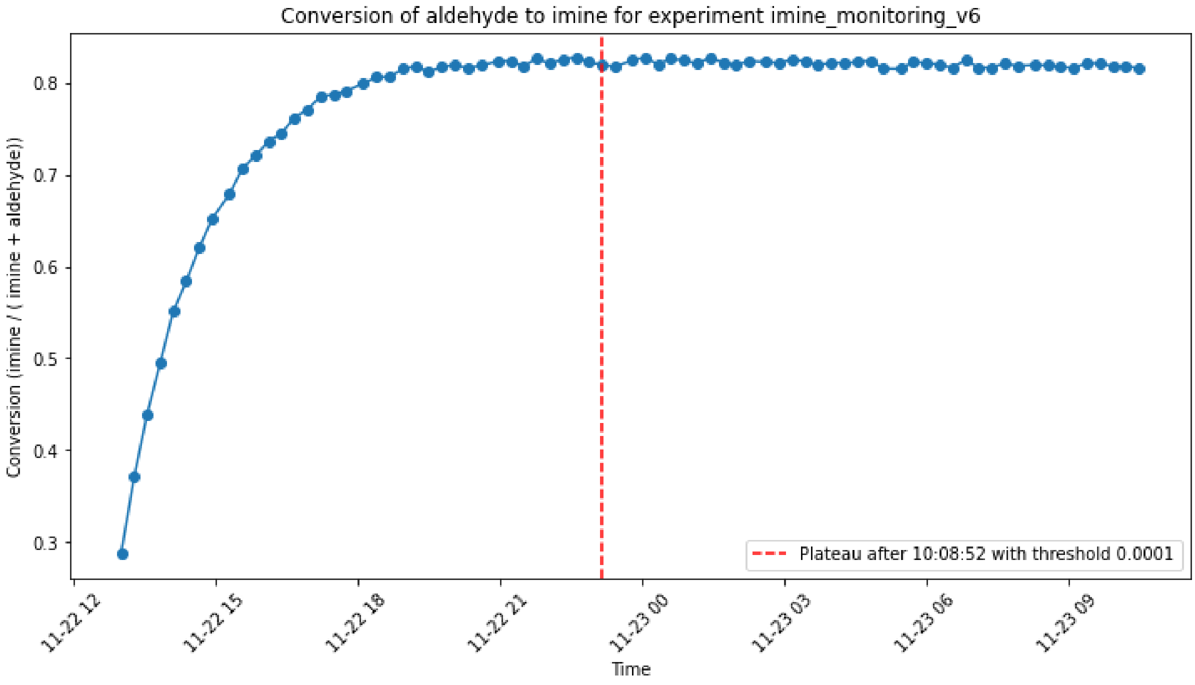

Figure S5: Following the conversion of the imine formation at 70 °C in $CHCl_3$ from **2** and 9-Anthracenecarboxaldehyde by dividing the integral under the imine product signal at 9.6 ppm by the sum of the integrals under the aldehyde substrate signal (at 10.5 ppm) and the imine signal (at 9.6 ppm).

5 Synthesis

5.1 Synthetic Outline

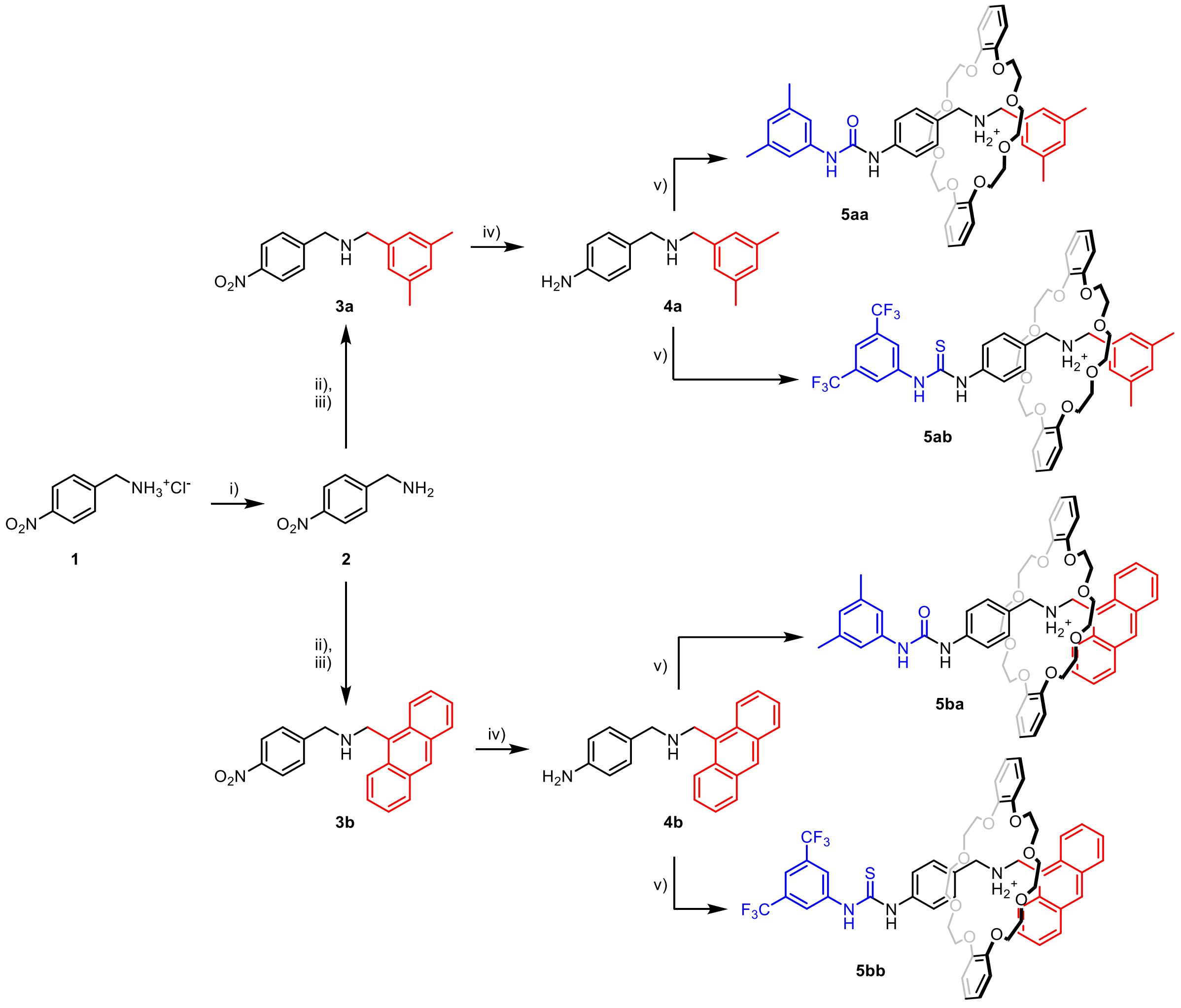

Scheme S1 – Divergent Synthesis of Rotaxanes. Reagents & Conditions:

- (i) **1** (1.0 eq.), NaOH (1.2 eq.), CH_2Cl_2:H_2O (1:1), r. t., 5 min, 83 %.
- (ii) **2** (1.0 eq.), 3,5-Dimethylbenzaldehyde or 9-Anthracenecarboxaldehyde (1.0 eq.), CH_2Cl_2:CH_3OH (1:1), r. t., max. 24 h, not isolated.
- (iii) $NaBH_4$ (2.0 eq.), CH_2Cl_2:CH_3OH (1:1), r. t., 2 h, not isolated, 67 % NMR yield (**3a**), 54 % NMR yield (**3b**).
- (iv) **3a** or **3b**, Zn dust (5.0 eq.), NH_4Cl (5.0 eq.), THF:CH_3OH (1:1), r. t., 15 h, stir at 1500 rpm, 66 % (**4a**), 60 % (**4b**).
- (v) **4a** or **4b**, DB24C8 (1.0 eq.), TFA (2.0 eq.), 3,5-Dimethylphenyl isocyanate or 3,5-Bis(trifluoromethyl)phenyl isothiocyanate (1.0 eq.), CH_2Cl_2, r. t., 16 h, 16 % (**5aa**), 63 % (**5ab**), 15 % (**5ba**), 8 % (**5bb**).

5.2 Automated Procedures & Characterisation Data

After optimisation of the reaction conditions for automated execution, the XDL protocols for the different steps were concatenated into one file for the formation of the diamine precursors and one file for the formation of the rotaxanes. This was practical because large batches (3 mmol / equiv.) of diamine were synthesized for convenience whereas the rotaxane formation was run on smaller scale (0.3 mmol / equiv.). The human readable output of the XDL Blueprints that are responsible for the different reaction steps are reported separately for clarity even though the XDL execution was not interrupted for the multi-step synthesis.

5.2.1 2

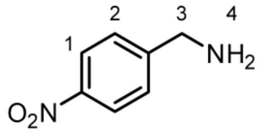

2

XDL #1.1, executed with graph #1

Reaction section

1) **Add 4-Nitrobenzylamine hydrochloride (1.0 mmol / equiv.) directly to separator.**
2) Add aqueous sodium hydroxide solution (1.2 mmol / equiv.) directly to separator.

Aqueous sodium hydroxide solution was prepared in a concentration of 1 mmol/L prior to the experiment.

3) Add water (3 mL / equiv.) directly to separator with stirring at 250 RPM.
4) Add dichloromethane (3 mL / equiv.) directly to separator with stirring at 250 RPM.

Workup section

1) Transfer all from separator directly to separator, flushing tubing after the transfer.
2) Add water (50 mL) directly to separator.
3) Add NaOH aqueous sodium hydroxide solution (20 mL) directly to separator.
4) Extract two-phase mixture in separator without adding solvent. Transfer waste phase (top) to vessel separator, and product phase (bottom) directly to buffer flask.
5) Extract contents of separator with dichloromethane (1 × 25 mL). Transfer waste phase (top) to waste, and product phase (bottom) directly to buffer flask.
6) Wash contents of buffer flask with brine (1 × 25 mL). Transfer waste phase (top) to waste, and product phase (bottom) through $MgSO_4$ to rotary evaporator.
7) Add water (5 mL) directly to solvent waste.
8) Add dichloromethane (20 mL) through $MgSO_4$ to rotary evaporator.
9) Add air (20 mL) through $MgSO_4$ to rotary evaporator.
10) Evaporate contents of rotary evaporator with pressure 500 mbar at temperature 40 °C for 15 min.
11) Add methanol (10 mL) directly to buffer flask.
12) Transfer 10 mL from buffer flask directly to solvent waste, flushing tubing after the transfer.
13) Add dichloromethane (10 mL) directly to buffer flask.
14) Transfer 10 mL from buffer flask directly to solvent waste, flushing tubing after the transfer.
15) Add water (60 mL) directly to separator.

16) Add dichloromethane (60 mL) directly to separator.
17) Stir separator for 60 s at 1000 RPM stopping stirring afterwards.
18) Transfer 130 mL from separator directly to solvent waste, flushing tubing after the transfer.
19) Clean separator with water (3 × solvent volume 10 mL).
20) Clean separator with water (1 × solvent volume 50 mL).
21) Clean separator with methanol (1 × solvent volume 50 mL).
22) Reset handling by cleaning the backbone with methanol (3 × 3 mL).

Yield: min. 83 %

^{1}H-NMR (600 MHz, $CDCl_3$): δ (ppm) 8.22 – 8.17 (m, 2H), 7.53 – 7.48 (m, 2H), 5.30 (s, 3H), 4.01 (s, 2H).

Data in accordance with literature.[S2]

5.2.2 3a

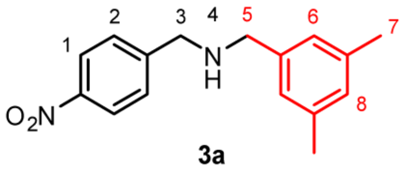

3a

XDL #1.1, executed with graph #1

Reaction section

1) Dissolve contents of rotary evaporator in dichloromethane (10 mL) over 20 min, stirring at 280 RPM.

2) Transfer 30 mL from rotary evaporator directly to reactor1, flushing tubing after the transfer.

3) Stir reactor1 for 12 h at 250 RPM stopping stirring afterwards.

4) Transfer 100 mL from reactor1 directly to reactor2, flushing tubing after the transfer.

5) Add methanol (5 mL / equiv.) directly to reactor2 over 60 min with stirring at 250 RPM.

6) Stir reactor2 for 2 h at 250 RPM stopping stirring afterwards.

7) Clean rotary evaporator with dichloromethane (2 × solvent volume 25 mL).

Workup section

1) Transfer all from separator directly to separator, flushing tubing after the transfer.
2) Add water (50 mL) directly to separator.
3) Add NaOH aqueous sodium hydroxide solution (20 mL) directly to separator.
4) Extract two-phase mixture in separator without adding solvent. Transfer waste phase (top) to vessel separator, and product phase (bottom) directly to buffer flask.
5) Extract contents of separator with dichloromethane (1 × 25 mL). Transfer waste phase (top) to waste, and product phase (bottom) directly to buffer flask.
6) Wash contents of buffer flask with brine (1 × 25 mL). Transfer waste phase (top) to waste, and product phase (bottom) through $MgSO_4$ to rotary evaporator.
7) Add water (5 mL) directly to solvent waste.
8) Add dichloromethane (20 mL) through $MgSO_4$ to rotary evaporator.
9) Add air (20 mL) through $MgSO_4$ to rotary evaporator.
10) Evaporate contents of rotary evaporator with pressure 500 mbar at temperature 40 °C for 15 min.
11) Add methanol (10 mL) directly to buffer flask.
12) Transfer 10 mL from buffer flask directly to solvent waste, flushing tubing after the transfer.
13) Add dichloromethane (10 mL) directly to buffer flask.
14) Transfer 10 mL from buffer flask directly to solvent waste, flushing tubing after the transfer.
15) Add water (60 mL) directly to separator.
16) Add dichloromethane (60 mL) directly to separator.
17) Stir separator for 60 s at 1000 RPM stopping stirring afterwards.
18) Transfer 130 mL from separator directly to solvent waste, flushing tubing after the transfer.
19) Clean separator with water (3 × solvent volume 10 mL).

20) Clean separator with water (1 × solvent volume 50 mL).
21) Clean separator with methanol (1 × solvent volume 50 mL).
22) Reset handling by cleaning the backbone with methanol (3 × 3 mL).

Yield: 67 % from quantitative NMR of crude mixture with 1,4-Bis(trimethylsilyl)benzene as internal standard. Product was carried forward without purification.

5.2.3 3b

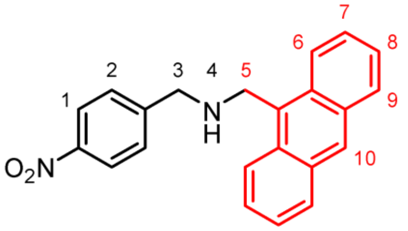

3b

XDL #1.2, executed with graph #1

Reaction section

1) Dissolve contents of rotary evaporator in dichloromethane (10 mL) over 20 min, stirring at 280 RPM.

2) Transfer 30 mL from rotary evaporator directly to reactor1, flushing tubing after the transfer.

3) Stir reactor1 for 12 h at 250 RPM stopping stirring afterwards.

4) Transfer 100 mL from reactor1 directly to reactor2, flushing tubing after the transfer.

5) Add methanol (5 mL / equiv.) directly to reactor2 over 60 min with stirring at 250 RPM.

6) Stir reactor2 for 2 h at 250 RPM stopping stirring afterwards.

7) Clean rotary evaporator with dichloromethane (2 × solvent volume 25 mL).

Workup section

1) Transfer all from separator directly to separator, flushing tubing after the transfer.
2) Add water (50 mL) directly to separator.
3) Add NaOH aqueous sodium hydroxide solution (20 mL) directly to separator.
4) Extract two-phase mixture in separator without adding solvent. Transfer waste phase (top) to vessel separator, and product phase (bottom) directly to buffer flask.
5) Extract contents of separator with dichloromethane (1 × 25 mL). Transfer waste phase (top) to waste, and product phase (bottom) directly to buffer flask.
6) Wash contents of buffer flask with brine (1 × 25 mL). Transfer waste phase (top) to waste, and product phase (bottom) through $MgSO_4$ to rotary evaporator.
7) Add water (5 mL) directly to solvent waste.
8) Add dichloromethane (20 mL) through $MgSO_4$ to rotary evaporator.
9) Add air (20 mL) through $MgSO_4$ to rotary evaporator.
10) Evaporate contents of rotary evaporator with pressure 500 mbar at temperature 40 °C for 15 min.
11) Add methanol (10 mL) directly to buffer flask.
12) Transfer 10 mL from buffer flask directly to solvent waste, flushing tubing after the transfer.
13) Add dichloromethane (10 mL) directly to buffer flask.
14) Transfer 10 mL from buffer flask directly to solvent waste, flushing tubing after the transfer.
15) Add water (60 mL) directly to separator.
16) Add dichloromethane (60 mL) directly to separator.
17) Stir separator for 60 s at 1000 RPM stopping stirring afterwards.

18) Transfer 130 mL from separator directly to solvent waste, flushing tubing after the transfer.
19) Clean separator with water (3 × solvent volume 10 mL).
20) Clean separator with water (1 × solvent volume 50 mL).
21) Clean separator with methanol (1 × solvent volume 50 mL).
22) Reset handling by cleaning the backbone with methanol (3 × 3 mL).

Yield: 54 % from quantitative NMR of crude mixture with 1,4-Bis(trimethylsilyl)benzene as internal standard. Product was carried forward without purification.

5.2.4 4a

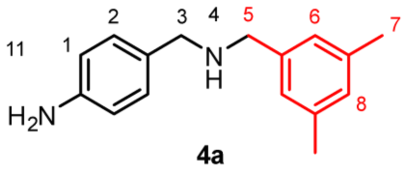

4a

XDL #1.1, executed with graph #1

Reaction section

1) **Add Zinc (17.5 mmol) directly to reactor3.**
2) **Add NH_4Cl (17.5 mmol) directly to reactor3.**
3) Dissolve contents of rotary evaporator in tetrahydrofuran (10 mL) over 5 min, stirring at 280 RPM.
4) Transfer 20 mL from rotary evaporator directly to reactor3, flushing tubing after the transfer.
5) Stir reactor3 for 12 h at 1500 RPM stopping stirring afterwards.
6) Clean rotary evaporator with tetrahydrofuran (2 × solvent volume 25 mL) stirring for 60 s at 150 RPM.
7) Clean reactor1 with dichloromethane (2 × solvent volume 20 mL) with drying if possible, stirring for 60 s at 250 RPM.
8) Transfer 20 mL from reactor3 directly to separator, flushing tubing after the transfer.

Workup section

1) Transfer all from separator directly to separator, flushing tubing after the transfer.
2) Add water (50 mL) directly to separator.
3) Add NaOH aqueous sodium hydroxide solution (20 mL) directly to separator.
4) Extract two-phase mixture in separator without adding solvent. Transfer waste phase (top) to vessel separator, and product phase (bottom) directly to buffer flask.
5) Extract contents of separator with dichloromethane (1 × 25 mL). Transfer waste phase (top) to waste, and product phase (bottom) directly to buffer flask.
6) Wash contents of buffer flask with brine (1 × 25 mL). Transfer waste phase (top) to waste, and product phase (bottom) through $MgSO_4$ to rotary evaporator.
7) Add water (5 mL) directly to solvent waste.
8) Add dichloromethane (20 mL) through $MgSO_4$ to rotary evaporator.
9) Add air (20 mL) through $MgSO_4$ to rotary evaporator.
10) Evaporate contents of rotary evaporator with pressure 500 mbar at temperature 40 °C for 15 min.
11) Add methanol (10 mL) directly to buffer flask.
12) Transfer 10 mL from buffer flask directly to solvent waste, flushing tubing after the transfer.
13) Add dichloromethane (10 mL) directly to buffer flask.
14) Transfer 10 mL from buffer flask directly to solvent waste, flushing tubing after the transfer.
15) Add water (60 mL) directly to separator.
16) Add dichloromethane (60 mL) directly to separator.
17) Stir separator for 60 s at 1000 RPM stopping stirring afterwards.
18) Transfer 130 mL from separator directly to solvent waste, flushing tubing after the transfer.
19) Clean separator with water (3 × solvent volume 10 mL).

20) Clean separator with water (1 × solvent volume 50 mL).
21) Clean separator with methanol (1 × solvent volume 50 mL).
22) Reset handling by cleaning the backbone with methanol (3 × 3 mL).

Yield: 66 %. Product was carried forward without purification.

^{1}H-NMR (600 MHz, $CDCl_3$): *δ* (ppm) 7.14 (d, *J* = 8.1 Hz, 2H, H_2), 6.94 (s, 2H, H_6), 6.88 (s, 1H, H_8), 6.66 (d, *J* = 7.9 Hz, 2H, H_1), 3.73 – 3.69 (m, 4H, $H_{3,5}$), 3.61 (br. s, 2H, H_{11}), 2.30 (s, 6H, H_7).

^{13}C NMR (151 MHz, $CDCl_3$): δ (ppm) 145.57, 139.65, 138.07, 129.70, 129.65, 128.81, 126.29, 115.26, 52.80, 52.66, 21.40.

HRMS-ESI (m/z) found 241.1702; [M+H] ($C_{16}H_{20}N_2H^+$) requires 241.1705.

5.2.5 4b

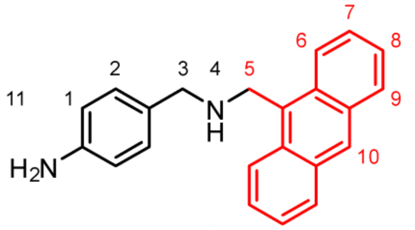

4b

XDL #1.2, executed with graph #1

Reaction section

1) **Add Zinc (17.5 mmol) directly to reactor3.**
2) **Add NH4Cl (17.5 mmol) directly to reactor3.**
3) Dissolve contents of rotary evaporator in tetrahydrofuran (10 mL) over 5 min, stirring at 280 RPM.
4) Transfer 20 mL from rotary evaporator directly to reactor3, flushing tubing after the transfer.
5) Stir reactor3 for 12 h at 1500 RPM stopping stirring afterwards.
6) Clean rotary evaporator with tetrahydrofuran (2 × solvent volume 25 mL) stirring for 60 s at 150 RPM.
7) Clean reactor1 with dichloromethane (2 × solvent volume 20 mL) with drying if possible, stirring for 60 s at 250 RPM.
8) Transfer 20 mL from reactor3 directly to separator, flushing tubing after the transfer.

Workup section

1) Transfer all from separator directly to separator, flushing tubing after the transfer.
2) Add water (50 mL) directly to separator.
3) Add NaOH aqueous sodium hydroxide solution (20 mL) directly to separator.
4) Extract two-phase mixture in separator without adding solvent. Transfer waste phase (top) to vessel separator, and product phase (bottom) directly to buffer flask.
5) Extract contents of separator with dichloromethane (1 × 25 mL). Transfer waste phase (top) to waste, and product phase (bottom) directly to buffer flask.
6) Wash contents of buffer flask with brine (1 × 25 mL). Transfer waste phase (top) to waste, and product phase (bottom) through $MgSO_4$ to rotary evaporator.
7) Add water (5 mL) directly to solvent waste.
8) Add dichloromethane (20 mL) through $MgSO_4$ to rotary evaporator.
9) Add air (20 mL) through $MgSO_4$ to rotary evaporator.
10) Evaporate contents of rotary evaporator with pressure 500 mbar at temperature 40 °C for 15 min.
11) Add methanol (10 mL) directly to buffer flask.
12) Transfer 10 mL from buffer flask directly to solvent waste, flushing tubing after the transfer.
13) Add dichloromethane (10 mL) directly to buffer flask.
14) Transfer 10 mL from buffer flask directly to solvent waste, flushing tubing after the transfer.
15) Add water (60 mL) directly to separator.
16) Add dichloromethane (60 mL) directly to separator.
17) Stir separator for 60 s at 1000 RPM stopping stirring afterwards.

18) Transfer 130 mL from separator directly to solvent waste, flushing tubing after the transfer.
19) Clean separator with water (3 × solvent volume 10 mL).
20) Clean separator with water (1 × solvent volume 50 mL).
21) Clean separator with methanol (1 × solvent volume 50 mL).
22) Reset handling by cleaning the backbone with methanol (3 × 3 mL).

Yield: 60 %. Product was carried forward as a crude without purification.

^{1}H-NMR (600 MHz, $CDCl_3$): *δ* (ppm) 8.39 (s, 1H, H_{10}), 8.23 (d, J = 8.8 Hz, 2H, H_6), 8.00 (d, *J* = 8.3 Hz, 2H, H_9), 7.52 – 7.42 (m, 4H, $H_{7,8}$), 7.23 (d, *J* = 8.3 Hz, 2H, H_2), 6.72 (d, *J* = 8.3 Hz, 2H, H_1), 4.67 (s, 2H, H_5), 3.94 (s, 2H, H_3), 3.65 (br. s, 2H, H_{11}).

^{13}C NMR (151 MHz, $CDCl_3$): δ 145.57, 131.98, 131.69, 130.52, 130.48, 129.64, 129.21, 127.23, 126.11, 125.02, 124.41, 115.31, 54.03, 44.85.

HRMS-ESI (m/z) found 313.1705; [M+H] ($C_{22}H_{20}N_2H^+$) requires 313.1705.

5.2.6 5aa

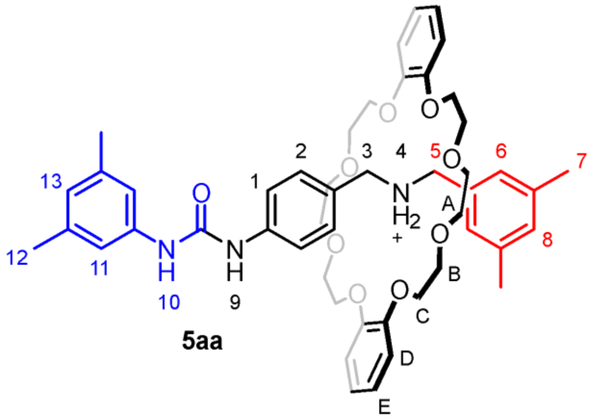

XDL 2.1, executed with graph #1

Reaction section (adapted from literature[S3])

1) Purify contents of rotary evaporator by preparative chromatography through column NP using the C815 flash chromatography system with detectors ['UV1']. Dissolve material in dichloromethane (10 mL) for 5 min and rinse rotary evaporator 1 times with dichloromethane (5 mL) before liquid loading onto the column. Collect fractions in left tray. Transfer product fraction to rotary evaporator afterwards. Elution gradient:

CV	-	-	Methanol	Dichloromethane
0	0	0	0	100
10	0	0	0	100
20	0	0	10	90
25	0	0	10	90

NP2: Biotage® Sfär Silica D column with 10 g normal phase silica, 15 mL column volume and 40 mL/min flow rate

UV1 detector: 220 nm

2) Evaporate contents of rotary evaporator with pressure 500 mbar at temperature 40 °C for 15 min.
3) Reset handling by cleaning the backbone with methanol (3 × 3 mL).
4) Reset handling by cleaning the backbone with water (3 × 3 mL).
5) Reset handling by cleaning the backbone with methanol (3 × 3 mL).
6) Reset handling by cleaning the backbone with CHCl3 (6 × 3 mL).
7) Analyse contents of flask $CHCl_3$ by moving 5 mL through NMR node, running an analytic experiment with parameters {'protocol': 'SHIM 1H SAMPLE', 'protocol options': {'Sample Reference': 7.26, 'Shim': 'Quick Shim All'}} and discarding the sample afterwards.

This step is used to shim the NMR spectrometer prior to the yield determination experiment.

8) Add NMR standard (0.1 mmol) directly to rotary evaporator with stirring at 250 RPM.

1,4-Bis(trimethylsilyl)benzene in deuterated chloroform (10 mmol/L) was used as an internal NMR standard for yield determination.

9) Stir rotary evaporator for 5 min at 250 RPM stopping stirring afterwards.
10) Analyse contents of rotary evaporator by moving 5 mL through NMR node, running an analytic experiment with default parameters, and returning the sample to rotary evaporator afterwards.

Default parameters on the NMR instrument are referring to a ^{1}H experiment with 16 scans.

11) Determine the ratio of the integrated NMR peaks from 2.5 to 2.2 ppm (product peak) and 0.5 to 0.1 ppm (reference peak) in most recent spectrum on NMR node and update product amount parameter with the result.
12) Add crown ether (1 mmol / equiv.) directly to rotary evaporator with stirring at 250 RPM.
13) Add TFA (2 mmol / equiv.) directly to rotary evaporator at default speed with stirring at 250 RPM.
14) Add isocyanate (1.2 mmol / equiv.) directly to rotary evaporator with stirring at 250 RPM.
15) Evaporate contents of rotary evaporator with pressure 500 mbar at temperature 40 °C for 15 min.
16) Add dichloromethane (10 mL) directly to rotary evaporator at default speed with stirring at 250 RPM.
17) Stir rotary evaporator for 16 h at 250 RPM stopping stirring afterwards.
18) Evaporate contents of rotary evaporator with pressure 500 mbar at temperature 40 °C for 15 min.
19) Evaporate contents of rotary evaporator with pressure 300 mbar at temperature 40 °C for 5 min.
20) Purify contents of rotary evaporator by preparative chromatography through column NP2 using the C815 flash chromatography system with detectors ['UV1']. Dissolve material in dichloromethane (10 mL) for 5 min and rinse rotary evaporator 1 times with dichloromethane (5 mL) before liquid loading onto the column. Collect fractions in left tray. Transfer product fraction to rotary evaporator afterwards. Elution gradient

CV	-	-	Methanol	Dichloromethane
0	0	0	0	100
10	0	0	0	100
20	0	0	10	90
25	0	0	10	90

NP2: Biotage® Sfär Silica D column with 10 g normal phase silica, 15 mL column volume and 40 mL/min flow rate

UV1 detector: 220 nm

21) Evaporate contents of rotary evaporator with pressure 500 mbar at temperature 40 °C for 15 min.
22) Purify contents of rotary evaporator by preparative chromatography through column SEC using the C815 flash chromatography system with detectors ['UV1']. Dissolve material in dichloromethane (10 mL) for 5 min and rinse rotary evaporator 1 times with dichloromethane (5 mL) before liquid loading onto the column. Collect fractions in right tray. Transfer product fraction to afterwards.

Elution gradient:

CV	-	-	Methanol	Dichloromethane
0	0	0	0	100
3	0	0	0	100

SEC: pressured cartridge with 100 mL column volume and 10 mL/min flow rate containing Bio-Beads S-X3 support beads as the stationary phase

UV1 detector: 220 nm

23) Shut down the platform.

Yield: 16 %

^{1}H NMR (600 MHz, $CDCl_3$) δ 10.12 (s, 1H, H_{10}), 9.59 (s, 1H, H_9), 7.78 (d, *J* = 8.9 Hz, 2H, H_1), 7.36 – 7.30 (m, 6H, $H_{4,2,11}$), 6.99 – 6.78 (m, 9H, $H_{D,E,8}$), 6.68 (s, 2H, H_6), 6.59 (s, 1H, H_{13}), 4.59 – 4.54 (m, 2H, H_3), 4.24 – 4.18 (m, 2H, H_5), 4.17 – 4.08 (m, 8H, H_C), 3.74 – 3.64 (m, 8H, H_B), 3.26 (m, 8H, H_A), 2.27 (s, 3H, H_{12}), 2.13 (s, 1H, H_7).

^{13}C NMR (151 MHz, $CDCl_3$) δ 154.42, 147.80, 142.96, 140.32, 138.20, 138.09, 131.68, 130.61, 130.30, 126.75, 123.65, 123.50, 122.17, 121.84, 118.64, 117.07, 113.29, 70.69, 70.24, 68.77, 52.87, 52.51, 21.62, 21.33.

HRMS-ESI (m/z) found 836.4484; [M+] ($C_{49}H_{62}N_3O_9^+$) requires 836.4481.

5.2.7 5ab

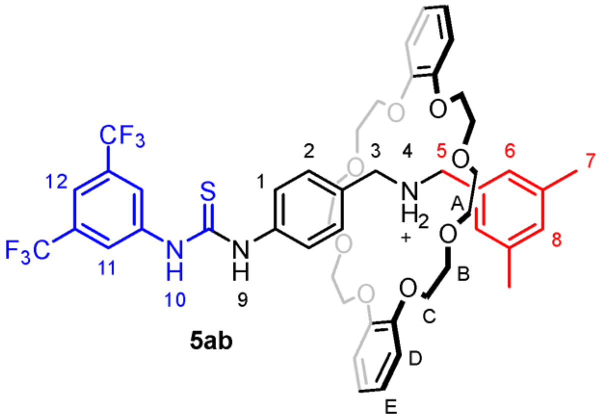

XDL #1.2, executed with graph #1

Yield: 63 %

^{1}H NMR (600 MHz, $CDCl_3$) δ 11.53 (s, 1H, H_{10}), 11.35 (s, 1H, H_9), 8.33 (s, 2H, H_{11}), 7.83 (d, *J* = 8.2 Hz, 2H, H_1), 7.55 (s, 1H, H_{12}), 7.44 (br. s, 2H, H_4) 7.40 (d, *J* = 7.9 Hz, 2H, H_2), 6.99 – 6.92 (m, 4H, H_D), 6.88 – 6.82 (m, 5H, $H_{E,8}$), 6.71 (s, 2H, H_6), 4.65 – 4.60 (m, 2H, H_3), 4.29 – 4.22 (m, 2H, H_5), 4.14 (m, 8H, H_C), 3.85 – 3.67 (m, 8H, H_B), 3.40 – 3.28 (m, 8H, H_A), 2.16 (s, 6H, H_7).

^{13}C NMR (151 MHz, $CDCl_3$) δ 180.95, 147.76, 141.83, 141.71, 138.30, 131.63, 131.25 (q, *J* = 33.5 Hz), 130.70, 129.80, 127.21, 126.70, 124.70, 124.32, 123.43 (q, *J* = 273.4 Hz), 122.27, 117.49, 113.36, 70.75, 70.26, 68.81, 52.79, 52.65, 21.37.

HRMS-ESI (m/z) found 960.3680; [M+] ($C_{49}H_{56}F_6N_3O_8S^+$) requires 960.3687

5.2.8 5ba

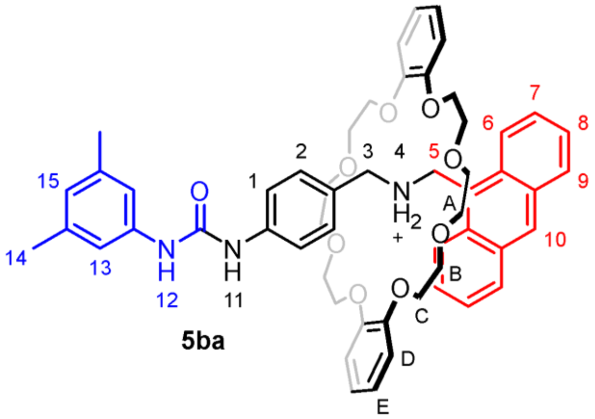

5ba

XDL #2.2, executed with graph #1

Yield: 15 %

^{1}H NMR (600 MHz, $CDCl_3$) δ 9.84 (s, 1H, H_{12}), 9.24 (s, 1H, H_{11}), 8.42 (d, *J* = 8.8 Hz, 2H, H_9), 8.08 (s, 1H, H_{10}), 7.80 (d, *J* = 8.4 Hz, 2H, H_6), 7.76 (d, *J* = 8.3 Hz, 2H, H_1), 7.63 (br. s, 2H, H_4), 7.51 (dd, *J* = 8.8, 6.4 Hz, 2H, H_8), 7.42 (dd, *J* = 8.4, 6.4 Hz, 2H, H_7), 7.31 (d, *J* = 8.3 Hz, 2H, H_2), 7.26 (s, 2H, H_{13}), 6.67 (m, 4H, H_E), 6.59 (s, 1H, H_{15}), 6.28 (m, 4H, H_D), 5.46 – 5.40 (m, 2H, H_5), 5.22 – 5.16 (m, 2H, H_3), 3.90 – 3.75 (m, 12H, $H_{C,B}$), 3.66 – 3.61 (m, 4H, $H_{B'}$), 3.56 (m, 4H, H_A), 3.42 – 3.36 (m, 4H, $H_{A'}$), 2.25 (s, 6H, H_{14}).

^{13}C NMR (151 MHz, $CDCl_3$) δ 154.33, 146.80, 141.90, 140.07, 138.15, 130.91, 130.87, 130.78, 129.59, 127.67, 127.03, 125.03, 124.49, 124.06, 123.75, 121.60, 121.56, 118.77, 117.00, 112.12, 71.17, 70.59, 68.15, 52.99, 45.26, 21.56.

HRMS-ESI (m/z) found 908.4483; [M+] ($C_{55}H_{62}N_3O_9^{+}$) requires 908.4481

5.2.9 5bb

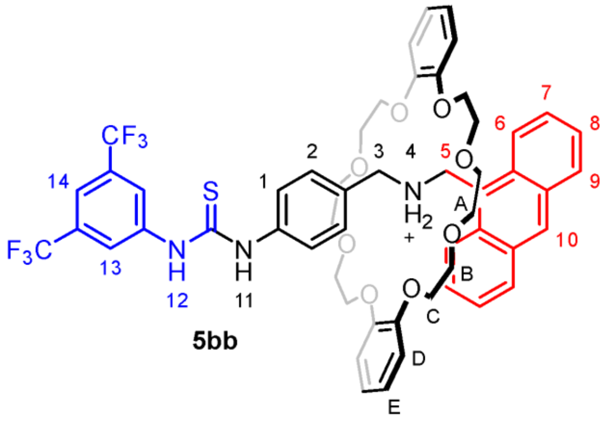

XDL #2.2, executed with graph #1

Yield: 8 %

^{1}H NMR (600 MHz, $CDCl_3$) δ 11.70 (s, 1H, $H_{11/12}$), 11.56 (s, 1H, $H_{12/11}$), 8.42 (d, *J* = 8.7 Hz, 2H, H_9), 8.33 (s, 2H, H_{13}), 8.09 (s, 1H, H_{10}), 7.92 (d, *J* = 8.1 Hz, 2H, H_1), 7.82 (d, *J* = 8.4 Hz, 2H, H_6), 7.69 (br. s, 2H, H_4), 7.59 – 7.50 (m, 3H, $H_{8,14}$), 7.48 – 7.37 (m, 4H, $H_{2,7}$), 6.69 (dt, *J* = 7.6, 3.8 Hz, 4H, H_E), 6.34 – 6.26 (m, 4H, H_D), 5.48 – 5.43 (m, 2H, H_5), 5.29 – 5.23 (m, 2H, H_3), 3.93 – 3.78 (m, 12H, $H_{C,B}$), 3.74 – 3.54 (m, 8H, $H_{B',A}$), 3.45 – 3.39 (m, 4H, $H_{A'}$).

^{13}C NMR (151 MHz, $CDCl_3$) δ 180.87, 146.78, 141.90, 141.15, 131.19 (q, *J* = 33.5 Hz), 130.92, 130.90, 130.87, 129.67, 127.94, 127.21, 127.13, 125.06, 124.53, 124.31, 123.95, 123.45 (q, *J* = 272.2 Hz), 121.66, 121.42, 117.40, 112.18, 71.24, 70.63, 68.20, 52.98, 45.39.

HRMS-ESI (m/z) found 1032.3684; [M+] ($C_{55}H_{56}F_6N_3O_8S^+$) requires 1032.3687

5.3 ^{1}H and ^{13}C NMR Spectra

5.3.1 ^{1}H and ^{13}C NMR Spectra of 4a

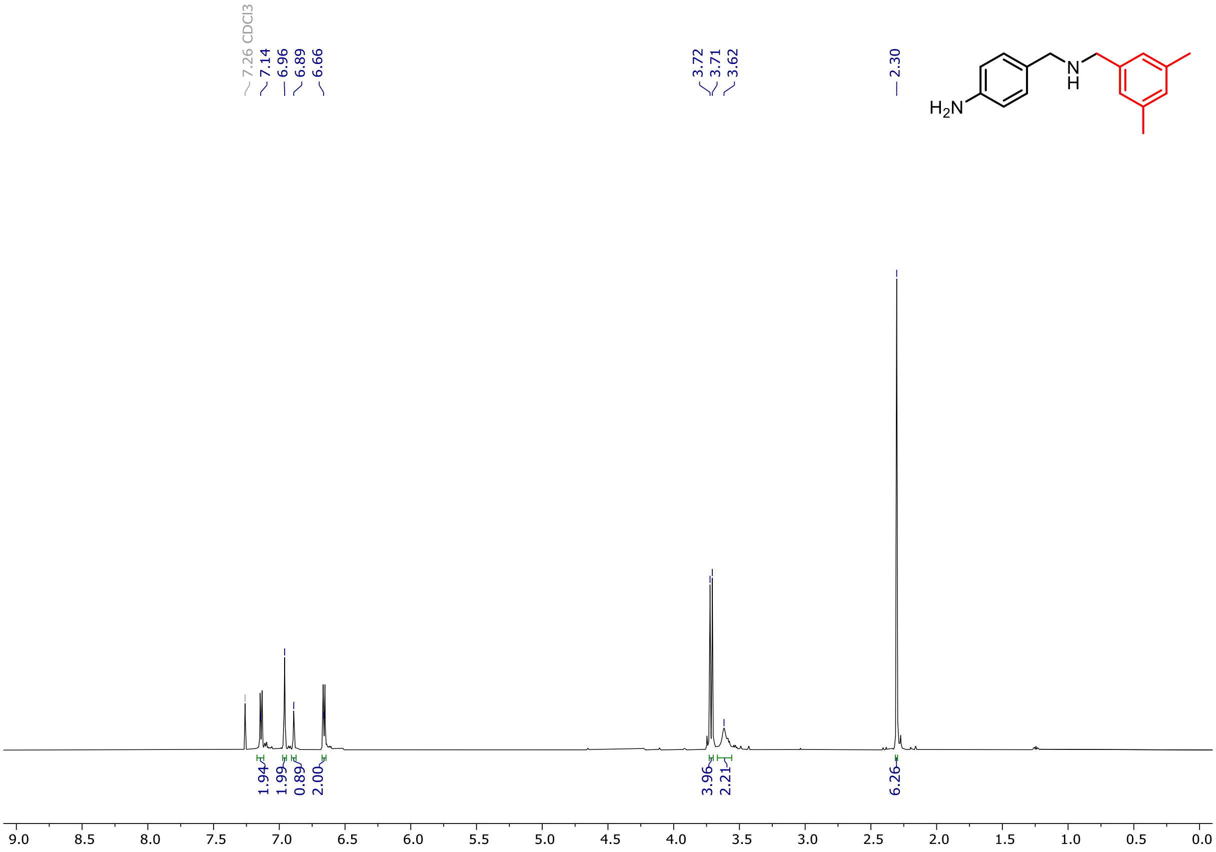

Spectrum S1 - ^{1}H NMR spectrum (600 MHz, $CDCl_3$, 300 K) of **4a**.

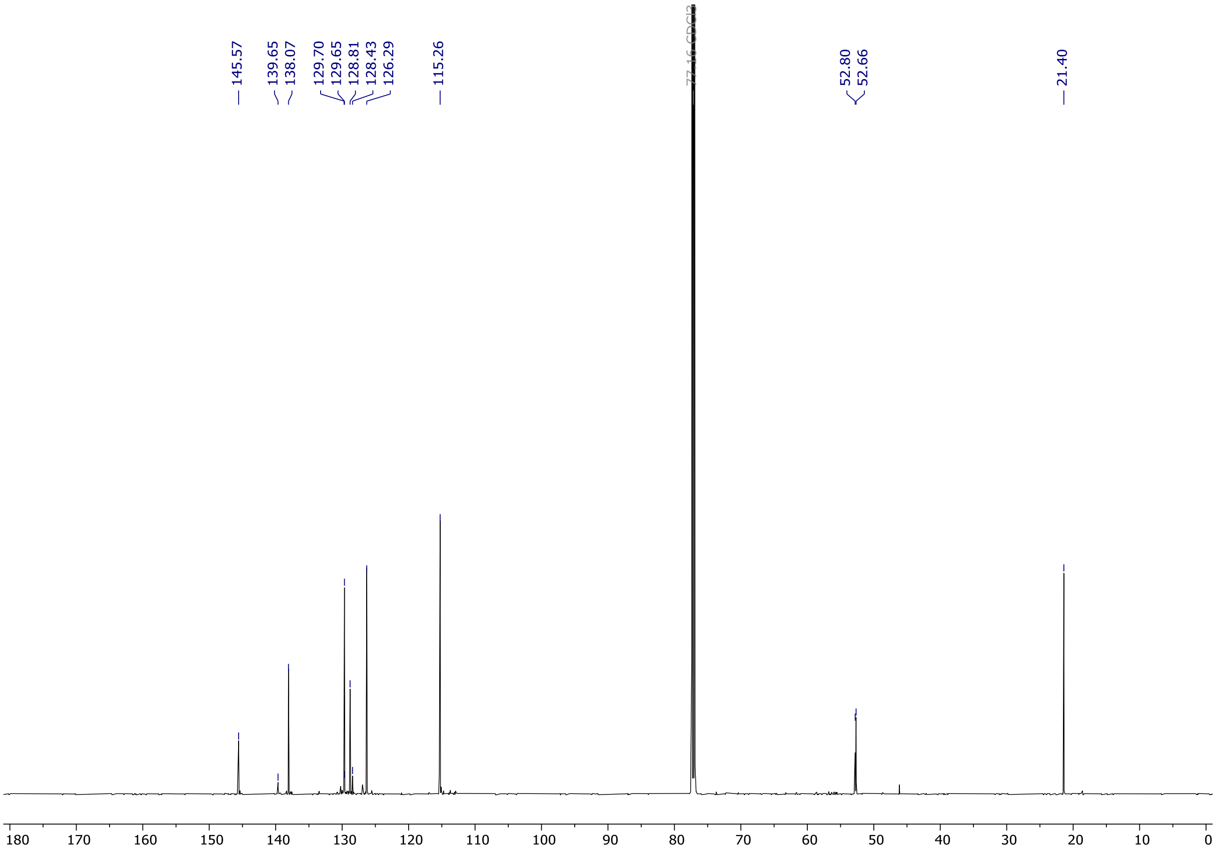

Spectrum S2 - ^{13}C NMR spectrum (151 MHz, $CDCl_3$, 300 K) of **4a**.

5.3.2 ^{1}H and ^{13}C NMR Spectra of 4b

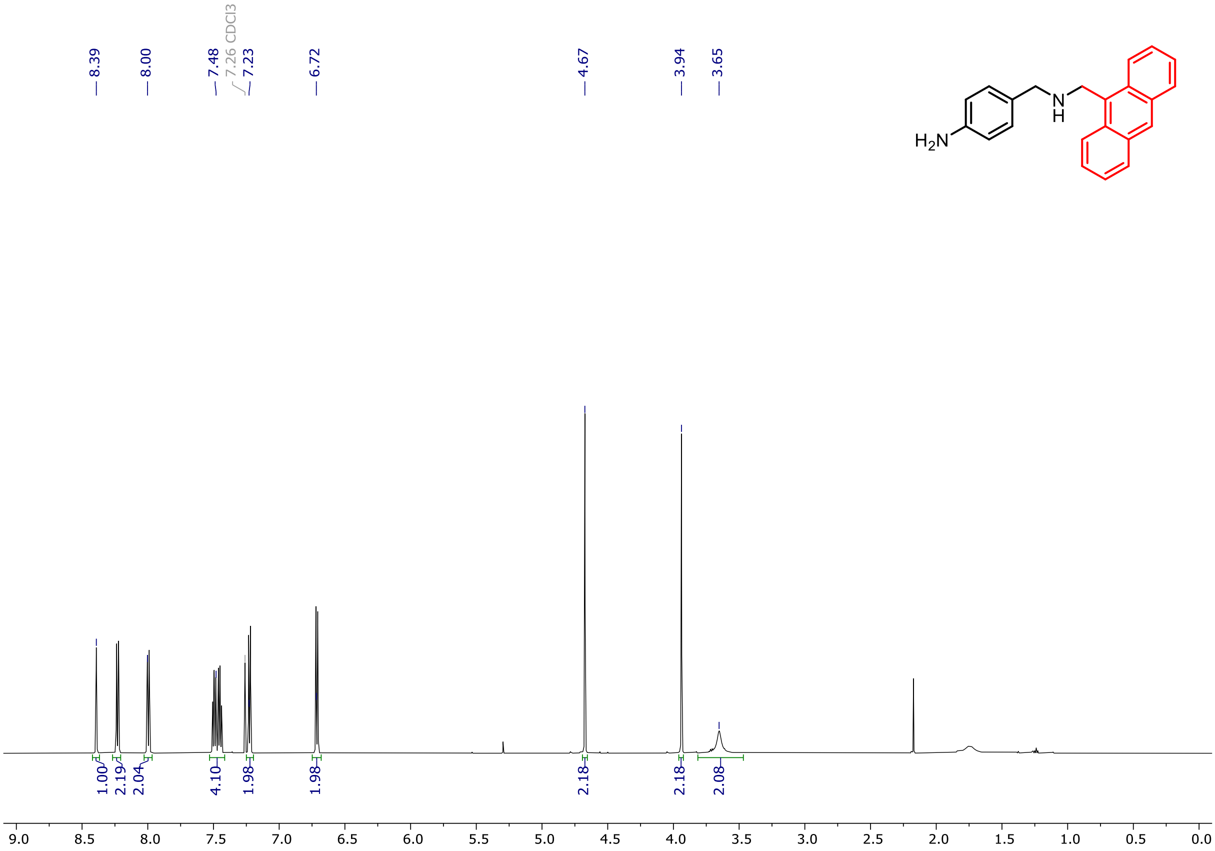

Spectrum S3 - ^{1}H NMR spectrum (600 MHz, $CDCl_3$, 300 K) of **4b**.

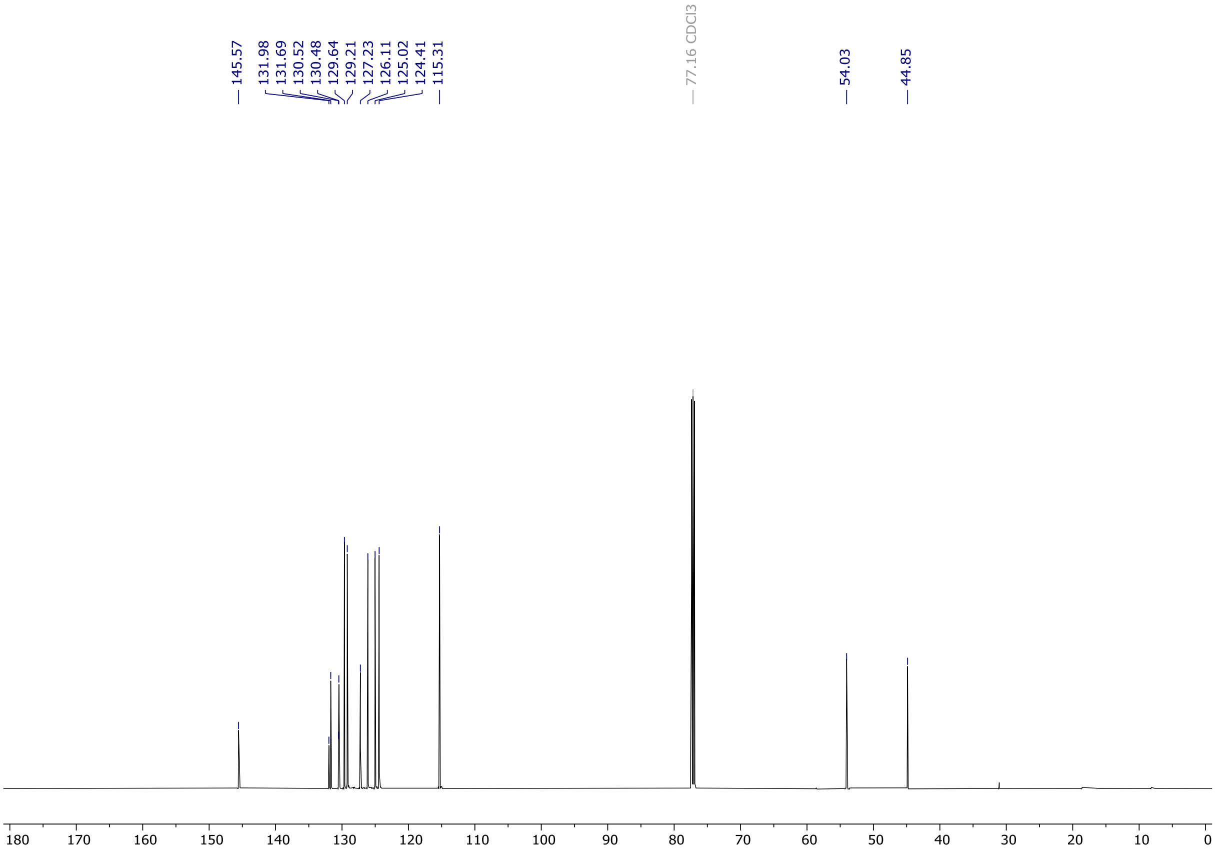

Spectrum S4 - ^{13}C NMR spectrum (151 MHz, $CDCl_3$, 300 K) of **4b**.

5.3.3 ^{1}H and ^{13}C NMR Spectra of 5aa

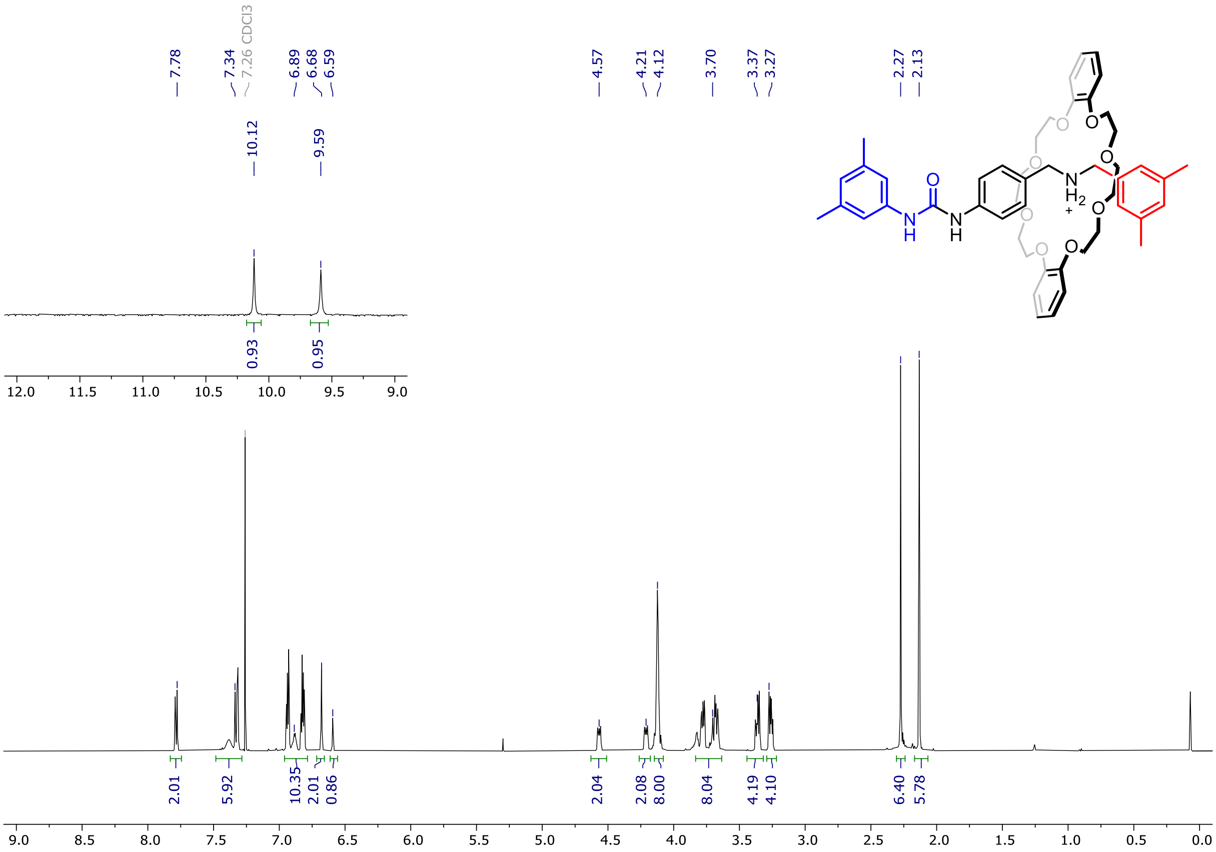

Spectrum S5 - ^{1}H NMR spectrum (600 MHz, $CDCl_3$, 300 K) of **5aa**.

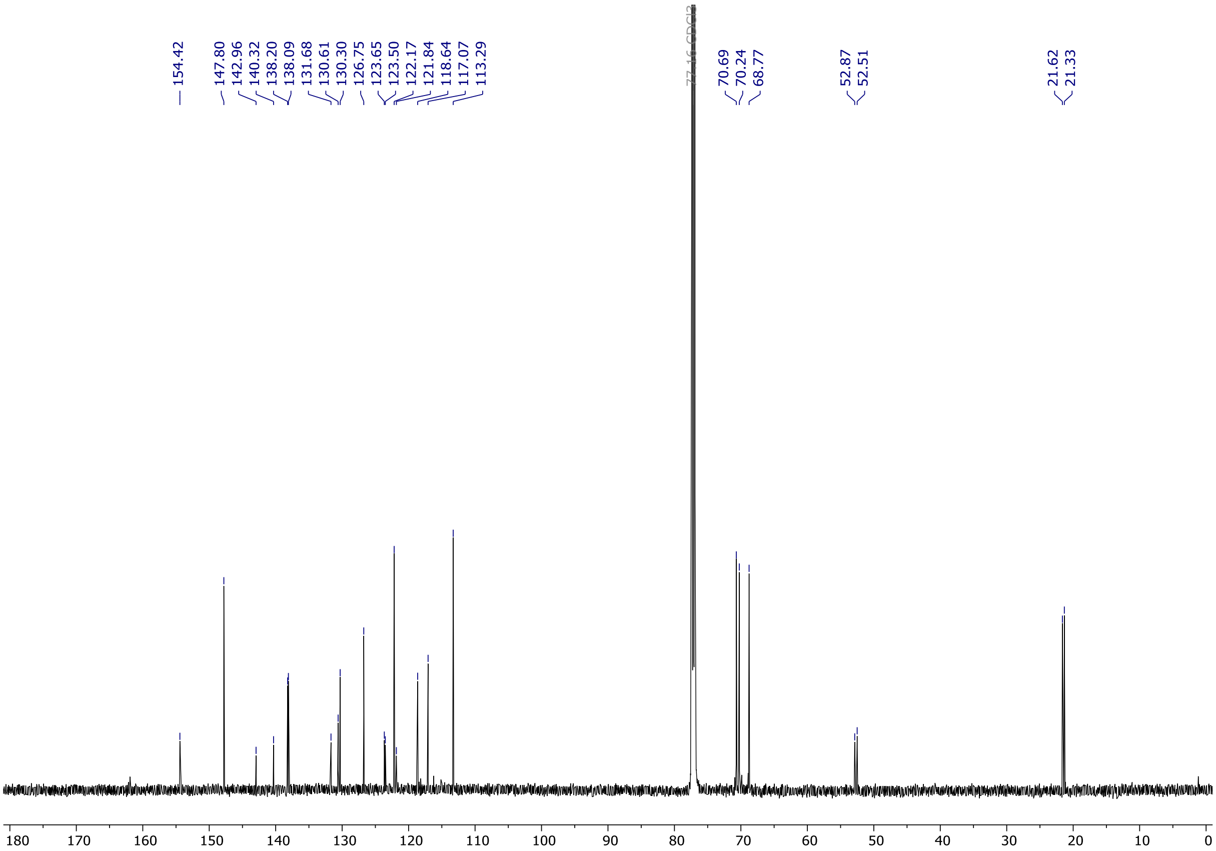

Spectrum S6 - ^{13}C NMR spectrum (151 MHz, $CDCl_3$, 300 K) of **5aa**.

5.3.4 ^{1}H and ^{13}C NMR Spectra of 5ab

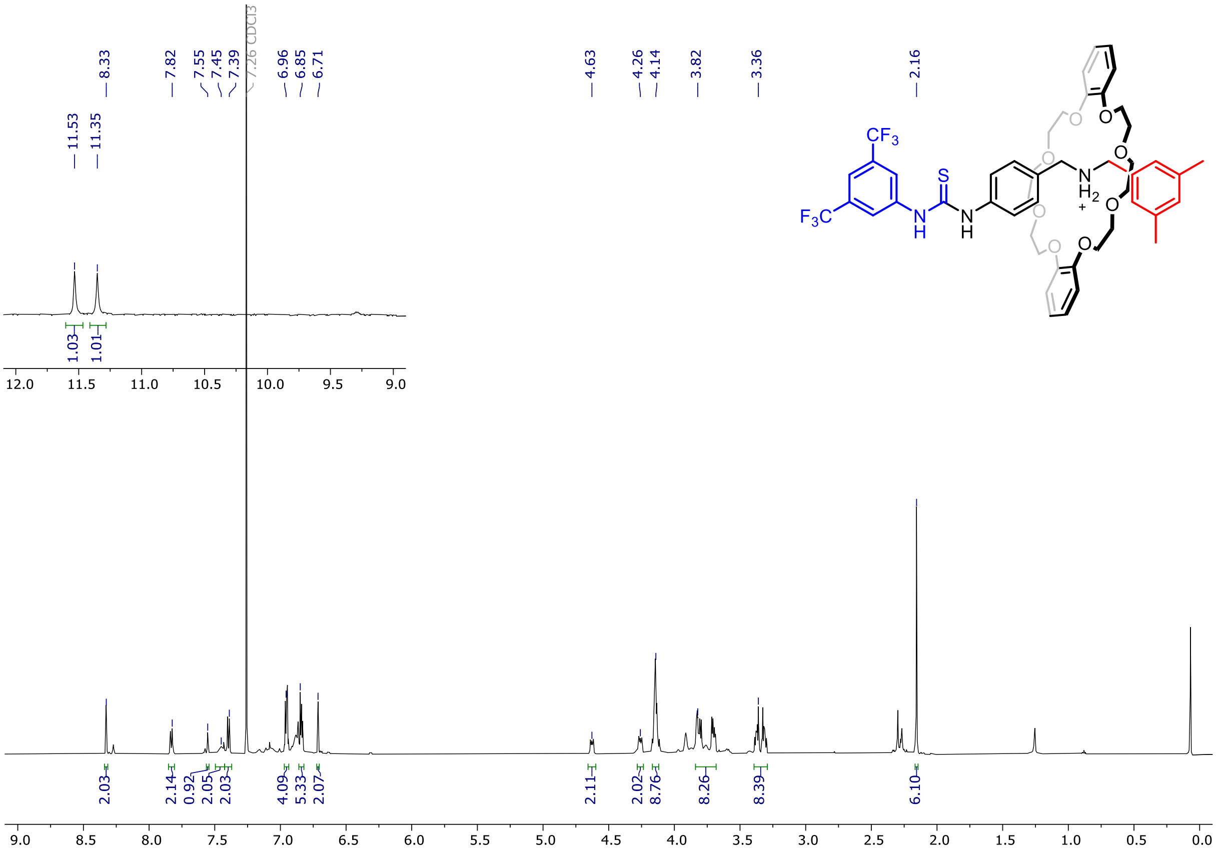

Spectrum S7 - ^{1}H NMR spectrum (600 MHz, $CDCl_3$, 300 K) of **5ab**.

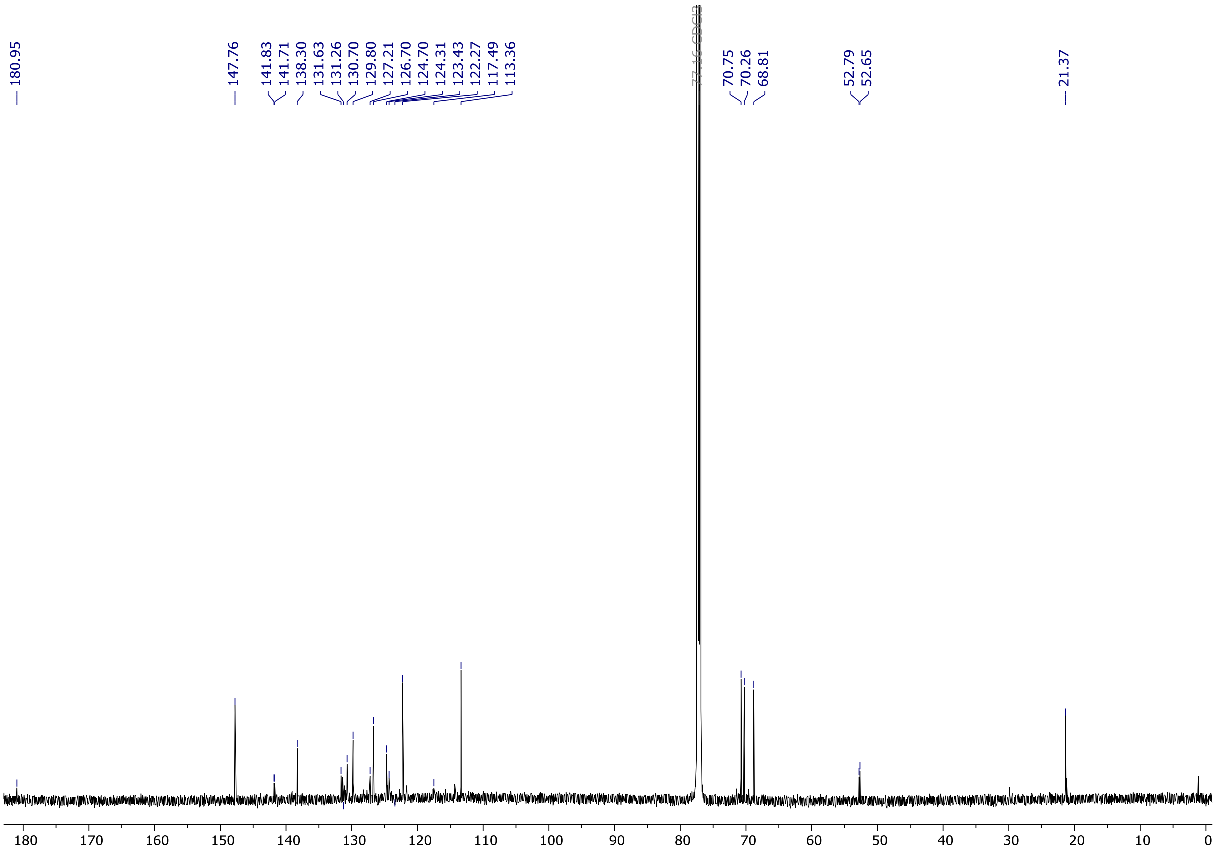

Spectrum S8 - ^{13}C NMR spectrum (151 MHz, $CDCl_3$, 300 K) of **5ab**.

5.3.5 ^{1}H and ^{13}C NMR Spectra of 5ba

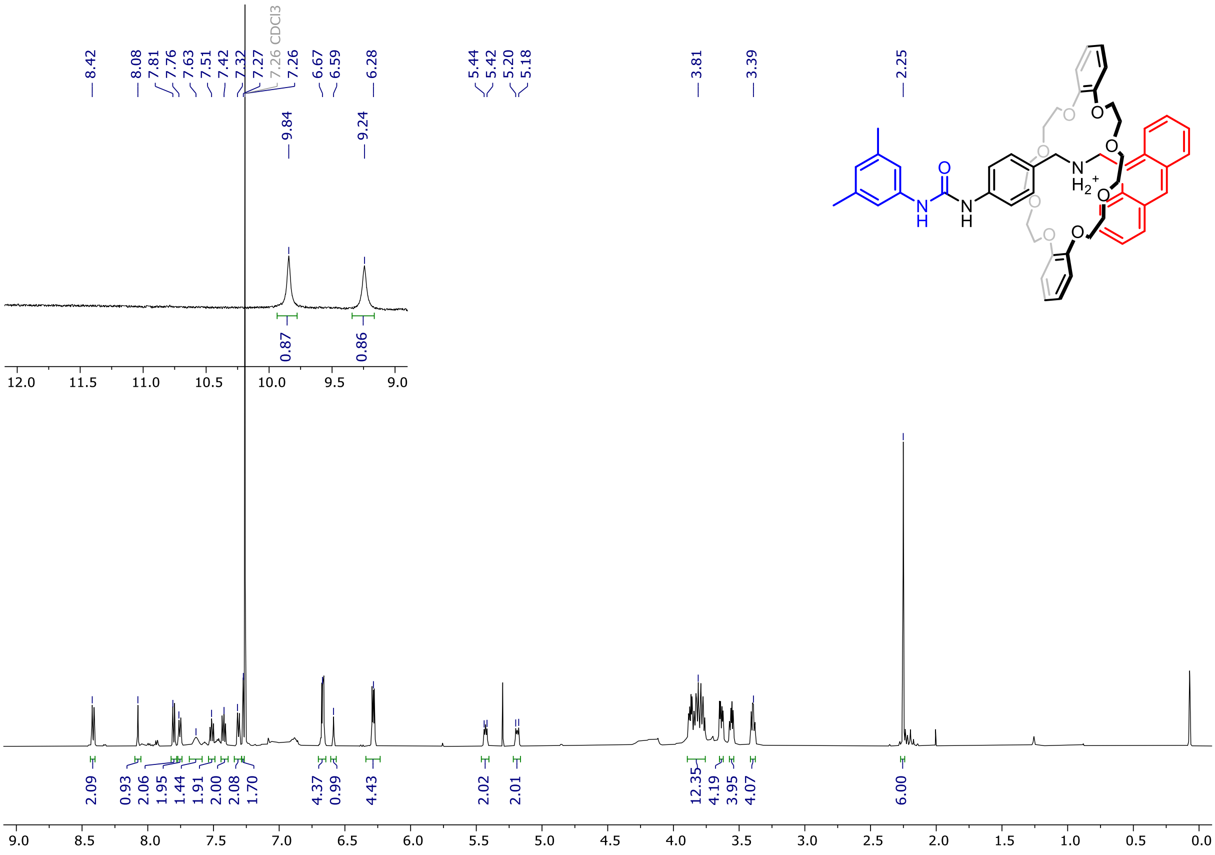

Spectrum S9 - ^{1}H NMR spectrum (600 MHz, $CDCl_3$, 300 K) of **5ba**.

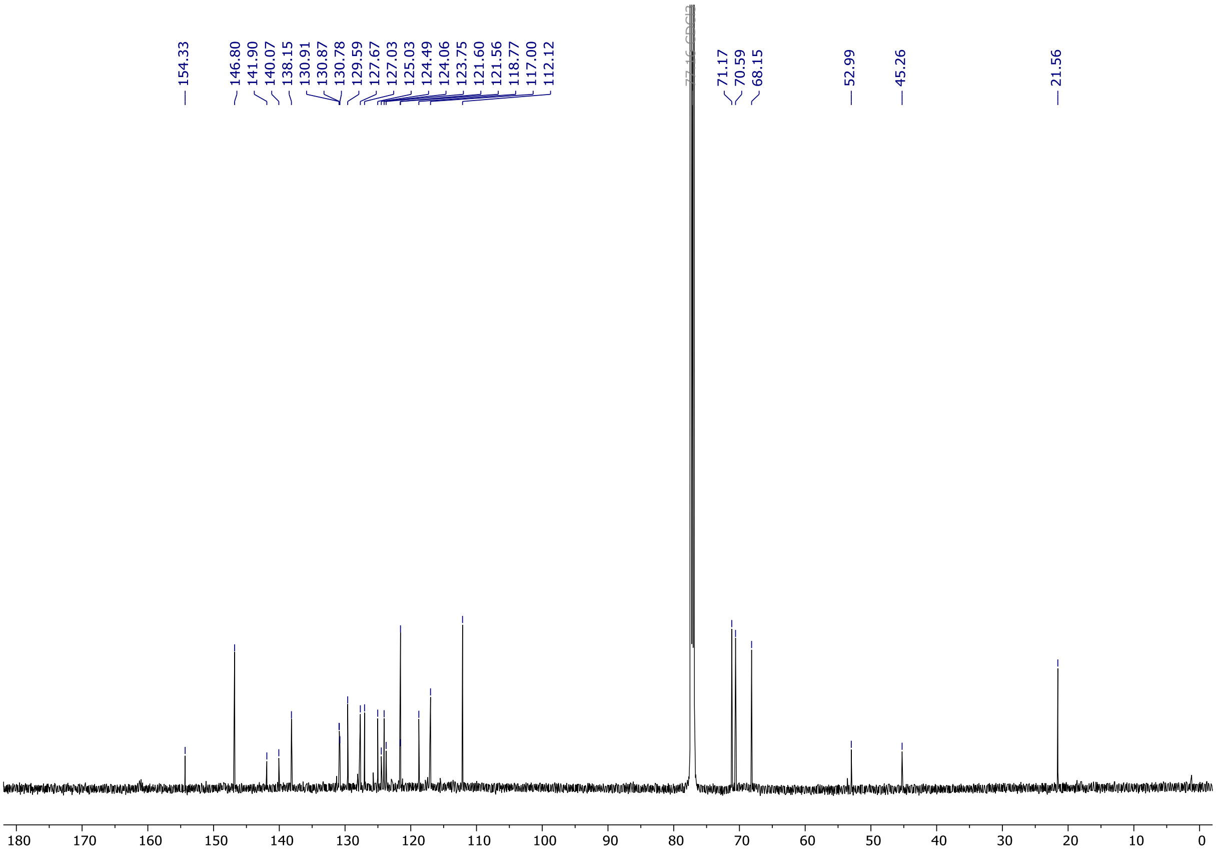

Spectrum S10 - ^{13}C NMR spectrum (151 MHz, $CDCl_3$, 300 K) of **5ba**.

5.3.6 ^{1}H and ^{13}C NMR Spectra of 5bb

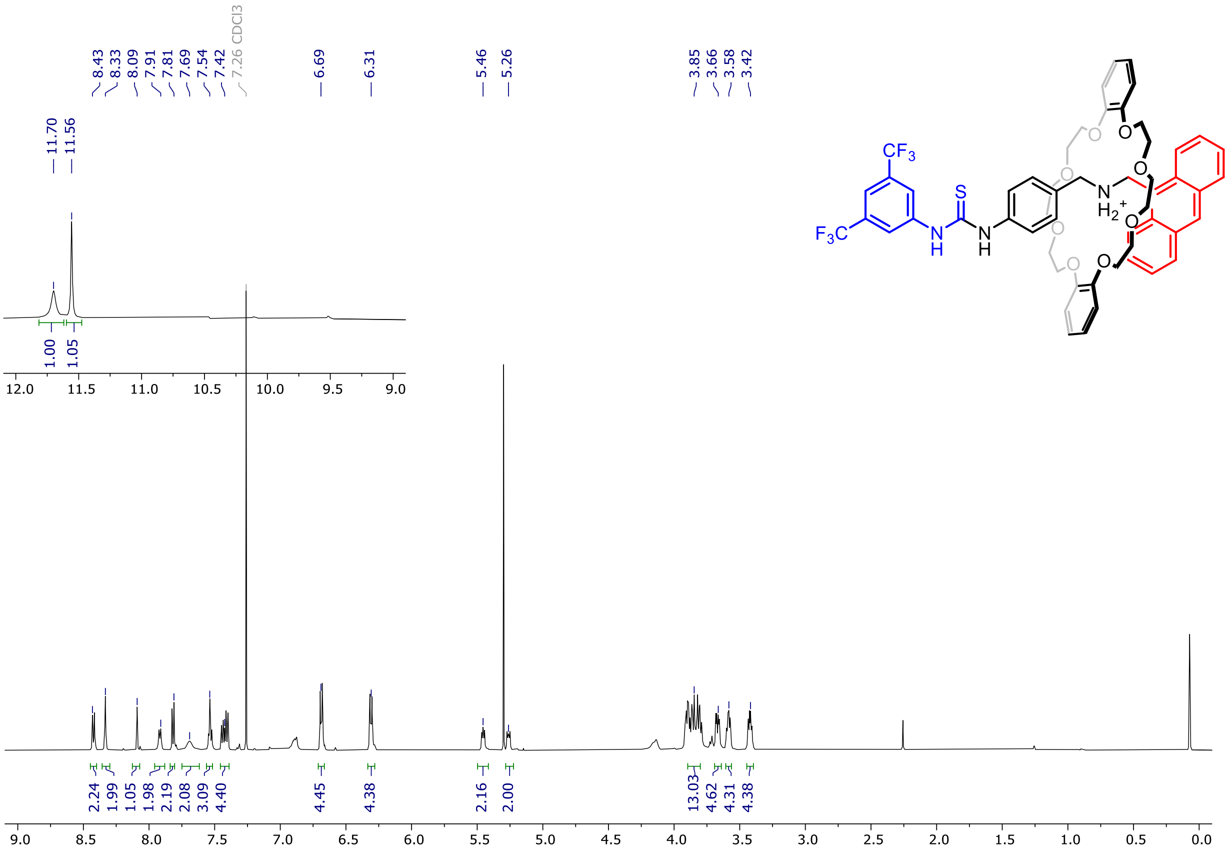

Spectrum S11 - ^{1}H NMR spectrum (600 MHz, $CDCl_3$, 300 K) of **5bb**.

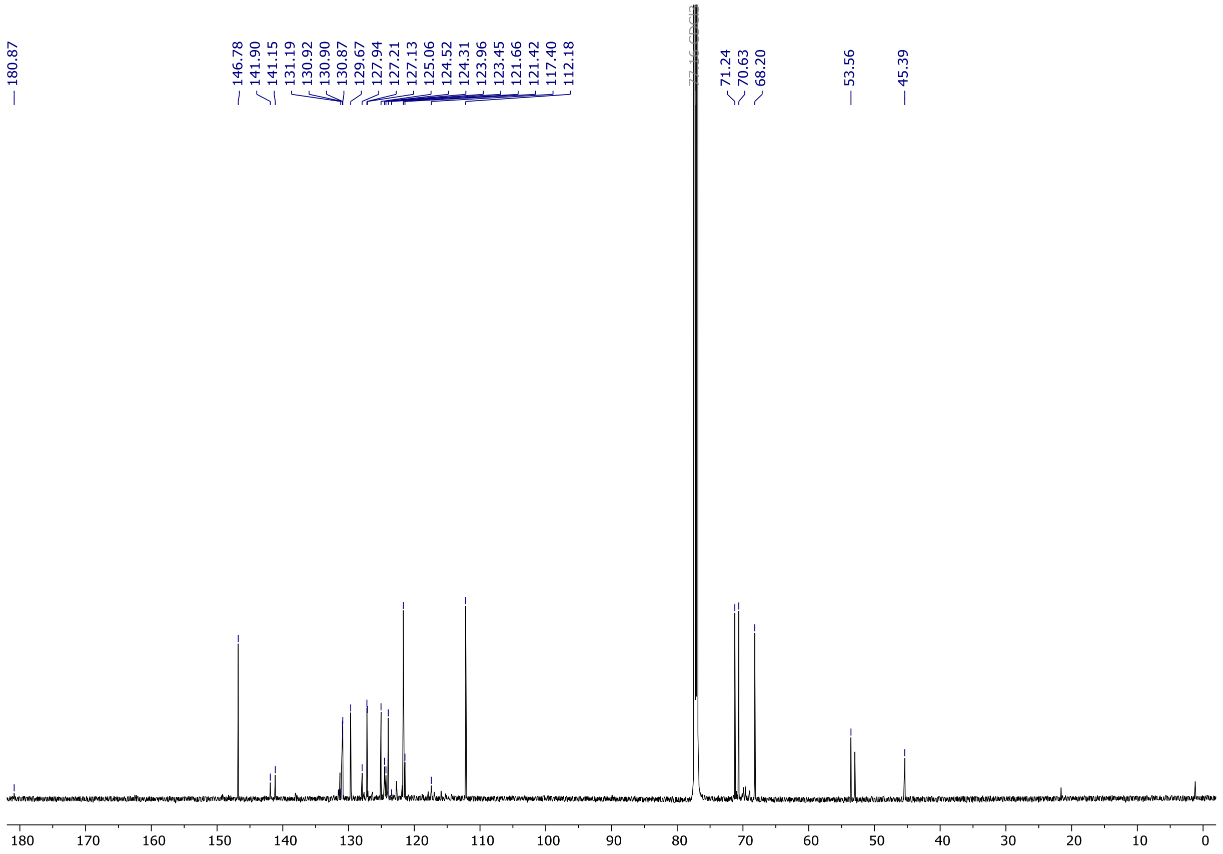

Spectrum S12 - ^{13}C NMR spectrum (151 MHz, $CDCl_3$, 300 K) of **5bb**.

6 Reliability & Reproducibility

The yields for the rotaxane synthesis on the Chemputer tend to be lower compared to manual synthesis, which we mainly attribute to the sample injection protocol upon purification. Due to the dead volume of the backbone tubing of the Chemputer, the minimal amount of solvent that is required to quantitatively transfer the crude sample from the rotary evaporator to the purification column tends to be in the same magnitude as the pump volume (5-10 mL). Therefore, the injection onto the column tends to be broad, leading to suboptimal separation upon elution. Furthermore, the loading method was optimised to minimise the injection of air which may disrupt the packed column. This issue is especially problematic with the size-exclusion beads which are sensitive to cracking. This means that the typical air flush that is appended to a liquid transfer on the Chemputer to ensure completeness of the operation is omitted. Consequently, a small amount of substance is inherently lost in the dead volume of the system before reaching the column. Further optimisation of the yield would be possible by switching to an injection system that is optimised for small volumes (for example a HPLC injection valve setup). In addition to that, the size of the cartridge that is used for size exclusion chromatography could be increased to enhance the purification performance and allow for a higher tolerance of air flush injection at the beginning of the gradient. Within this project we optimised the system for quick purification of multiple samples in sequence, to obtain small but pure amounts of every rotaxane for analysis.

In the manual synthesis, this bottleneck is avoided by dissolving the crude sample in a minimal amount of solvent (dichloromethane) and taking the solution up with a small syringe that was then used to directly inject the sample onto the column without any tubing involved.

Table S2 – Reproducibility and reliability of a Chemputer yields in comparison to conventional methods.

Compound	Manual (%)	Literature (%)	Average Chemputer (%) ± SD (%)	Samples
**2**	95	97[S4]	83 ± 15	9
**3a**	96	91[S5]	91 ± 11	2
**3b**	99	—	97 ± 2	4
**4a**	14	88*[S5]	60 ± 22	4
**4b**	19	—	50 ± 32	8
**5aa**	46	—	16	1
**5ab**	38	—	63	1
**5ba**	51	—	8	1
**5bb**	60	—	15	1

*Different experimental method was utilised.

7 References

S1. Rohrbach, S., Šiaučiulis, M., Chisholm, G., Pirvan, P.-A., Saleeb, M., Mehr, S.H.M., Trushina, E., Leonov, A.I., Keenan, G., Khan, A., et al. (2022). Digitization and validation of a chemical synthesis literature database in the ChemPU. Science *377*, 172–180. 10.1126/science.abo0058.

S2. Amberchan, G., Snelling, R.A., Moya, E., Landi, M., Lutz, K., Gatihi, R., and Singaram, B. (2021). Reaction of Diisobutylaluminum Borohydride, a Binary Hydride, with Selected Organic Compounds Containing Representative Functional Groups. J. Org. Chem. *86*, 6207–6227. 10.1021/acs.joc.0c03062.

S3. Cantrill, S.J., Stoddart, J.F., and White, A.J.P. (1999). A New Protocol for Rotaxane Synthesis. Tet. Lett. *40*, 3669–3672. 10.1016/S0040-4039(99)00555-9.

S4. Scheffelaar, R., Paravidino, M., Muilwijk, D., Lutz, M., Spek, A.L., De Kanter, F.J.J., Orru, R.V.A., and Ruijter, E. (2009). A Novel Three-Component Reaction toward Dihydrooxazolopyridines. Org. Lett. *11*, 125–128. 10.1021/ol802515v.

S5. Cheng, M., Liu, L., Cao, Y., Jiang, J., and Wang, L. (2016). A Phosphine Oxide Functional Group Based [2]Rotaxane That Operates as a Multistable Molecular Shuttle. ChemPhysChem *17*, 1835–1839. 10.1002/cphc.201501016.
